# Supplementary material for: Superior normalization using total protein for western blot analysis of human adipocytes
Source: PLoS One. 2025 Jul 22;20(7):e0328136. doi: 10.1371/journal.pone.0328136 (PMC12282925; doi:10.1371/journal.pone.0328136)
Supplement: S2 Table — (DOCX) [file pone.0328136.s002.docx]

**S2 Table.**

| **Sample** | **Concentration(µg/µL)** | **Loading (µL) needed for 10 µg** |
| --- | --- | --- |
| **BSA** | 2.966 | 3.37 |
| **BSA + wash x3** | 0.592 | 16.88 |
| **BSA free** | 0.496 | 20.14 |
| **BSA free + wash x3** | 0.400 | 24.97 |
