# Supplementary material for: Superior normalization using total protein for western blot analysis of human adipocytes
Source: PLoS One. 2025 Jul 22;20(7):e0328136. doi: 10.1371/journal.pone.0328136 (PMC12282925; doi:10.1371/journal.pone.0328136)
Supplement: S3 Table — (DOCX) [file pone.0328136.s003.docx]

**S3 Table.**

| **Clinical parameters** | **Individual 1** | **Individual 2** | **Individual 3** |
| --- | --- | --- | --- |
| **Depot** | OM | OM | OM |
| **Age** | 47 | 50 | 46 |
| **Sex** | Female | Female | Female |
| **BMI (kg/m^2^)** | 37.41 | 37.73 | 38.12 |
| **Fat (%)** | 48.3 | 45.4 | 43.2 |
| **Hip (cm)** | 122.5 | 120 | 123 |
| **Waist (cm)** | 111 | 102 | 95 |
| **WHR** | 0.91 | 0.85 | 0.77 |
| **Medication** | None | None | None |
| **Glucose** | 5.7 | 5.8 | 5.7 |
| **C-peptide** | 0.99 | 1 | 1 |
| **Insulin** | 10 | 12 | 13 |
