# Supplementary material for: Superior normalization using total protein for western blot analysis of human adipocytes
Source: PLoS One. 2025 Jul 22;20(7):e0328136. doi: 10.1371/journal.pone.0328136 (PMC12282925; doi:10.1371/journal.pone.0328136)
Supplement: S4 Table — (DOCX) [file pone.0328136.s004.docx]

**S4 Table.**

| Clinical Parameters | BSA | OBHI OM | OBHI SC | OBHI OM | OBHI SC | Dynamic range |
| --- | --- | --- | --- | --- | --- | --- |
| Depot | OM | OM | SC | OM | SC | OM |
| Age | 52 | 53 | 63 | 29 | 53 | 37 |
| Sex | Male | Female | Female | Female | Female | Female |
| BMI (kg/m^2^) | 38.51 | 38.38 | 42.05 | 34.05 | 33.94 | 43.47 |
| Fat (%) | 36.4 | 49 | 51.9 | 48.9 | 47.6 | 53.3 |
| Hip (cm) | 139 | 124 | 133 | 124 | 119 | 131 |
| Waist (cm) | 142 | 114 | 130 | 103 | 101 | 109 |
| WHR | 1.02 | 0.92 | 0.98 | 0.83 | 0.85 | 0.83 |
| Medication | Metformin, Xultrophy | losartan, imraldi, dymista | lamictal, eliqwas, atorvastatin | bricanyl | Levaxin | Brintellix |
| Glucose | 5.6 | 6.3 | 5.5 | 5.6 | 5.7 | 5.4 |
| C-peptide | 0.56 | 1.4 | 1.8 | 0.81 | 0.59 | 1.6 |
| Insulin | 12 | 24 | 42 | 14 | 8.5 | 30 |
