# Supplementary figures and images for: Superior normalization using total protein for western blot analysis of human adipocytes
Source: PLoS One. 2025 Jul 22;20(7):e0328136. doi: 10.1371/journal.pone.0328136 (PMC12282925; doi:10.1371/journal.pone.0328136)

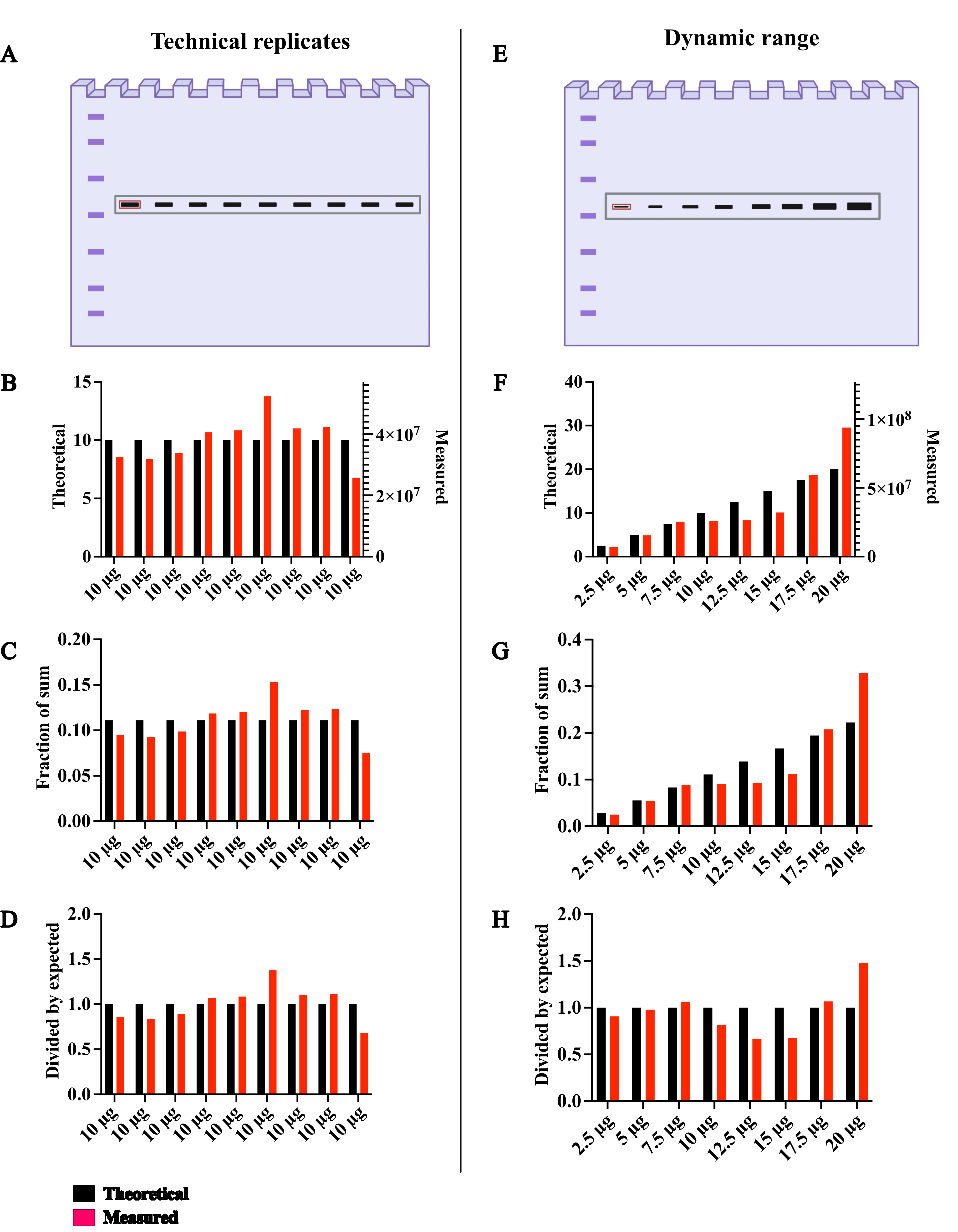

Supplement: S1 Fig — (A) Schematic for sum normalization of technical replicates, (B) theoretical and measured data, (C) fraction of sum, (D) divided by expected. (E) Schematic for sum normalization of dynamic range, (F) theoretical and measured data, (G) fraction of sum, (H) divided by expected. (TIF) [file pone.0328136.s005.tif]

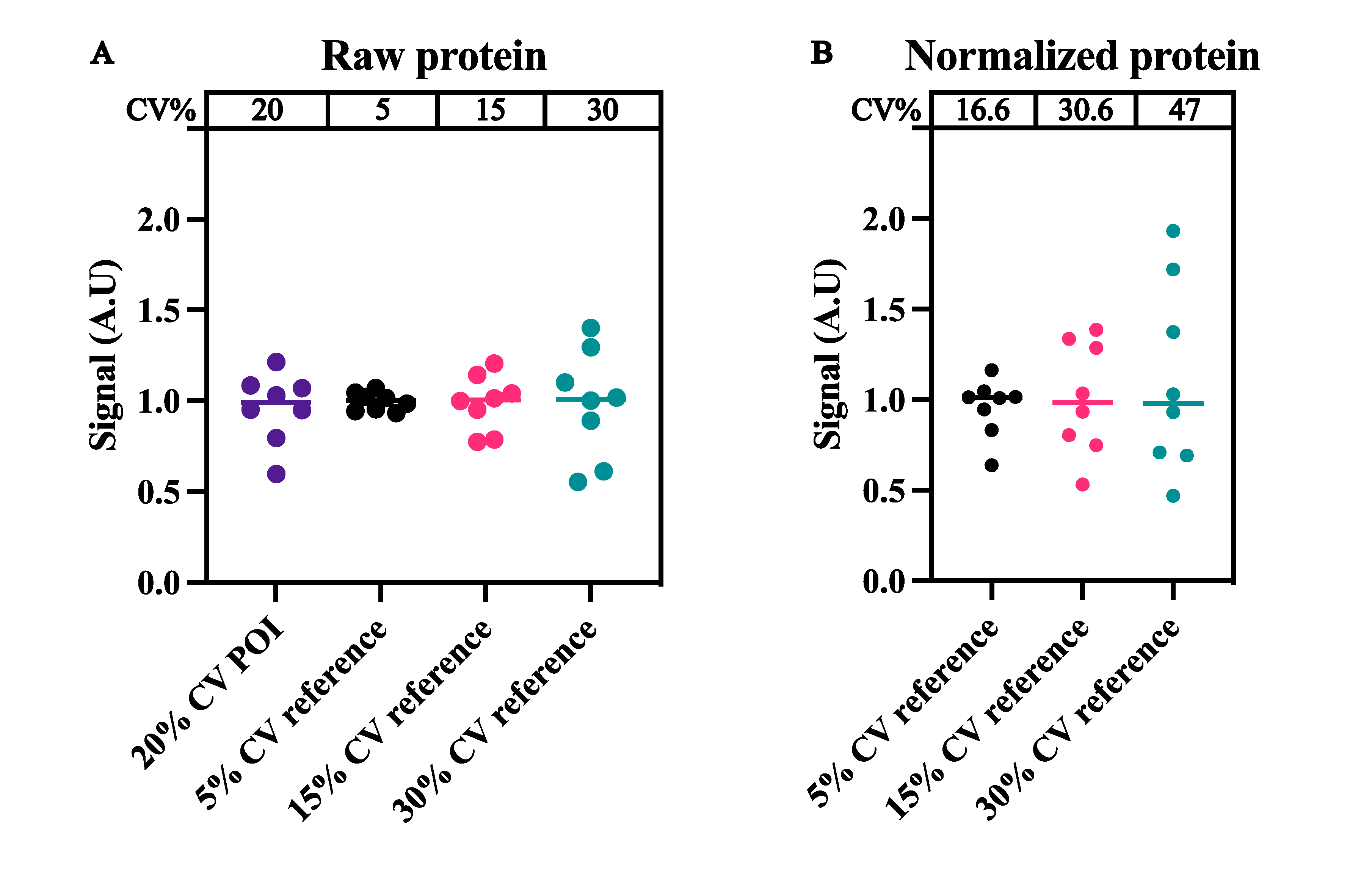

Supplement: S2 Fig — (A) Simulated raw signal intensities of a protein of interest (POI) and three different normalization references, each with a coefficient of variation (CV) of 5%, 15%, or 30%. (B) Normalized POI signal obtained by dividing the POI values by each respective normalization reference shown in (A). This demonstrates how increasing variability in the reference impacts the stability of the normalized signal, despite the POI having identical underlying variability in all cases. (TIF) [file pone.0328136.s006.tif]

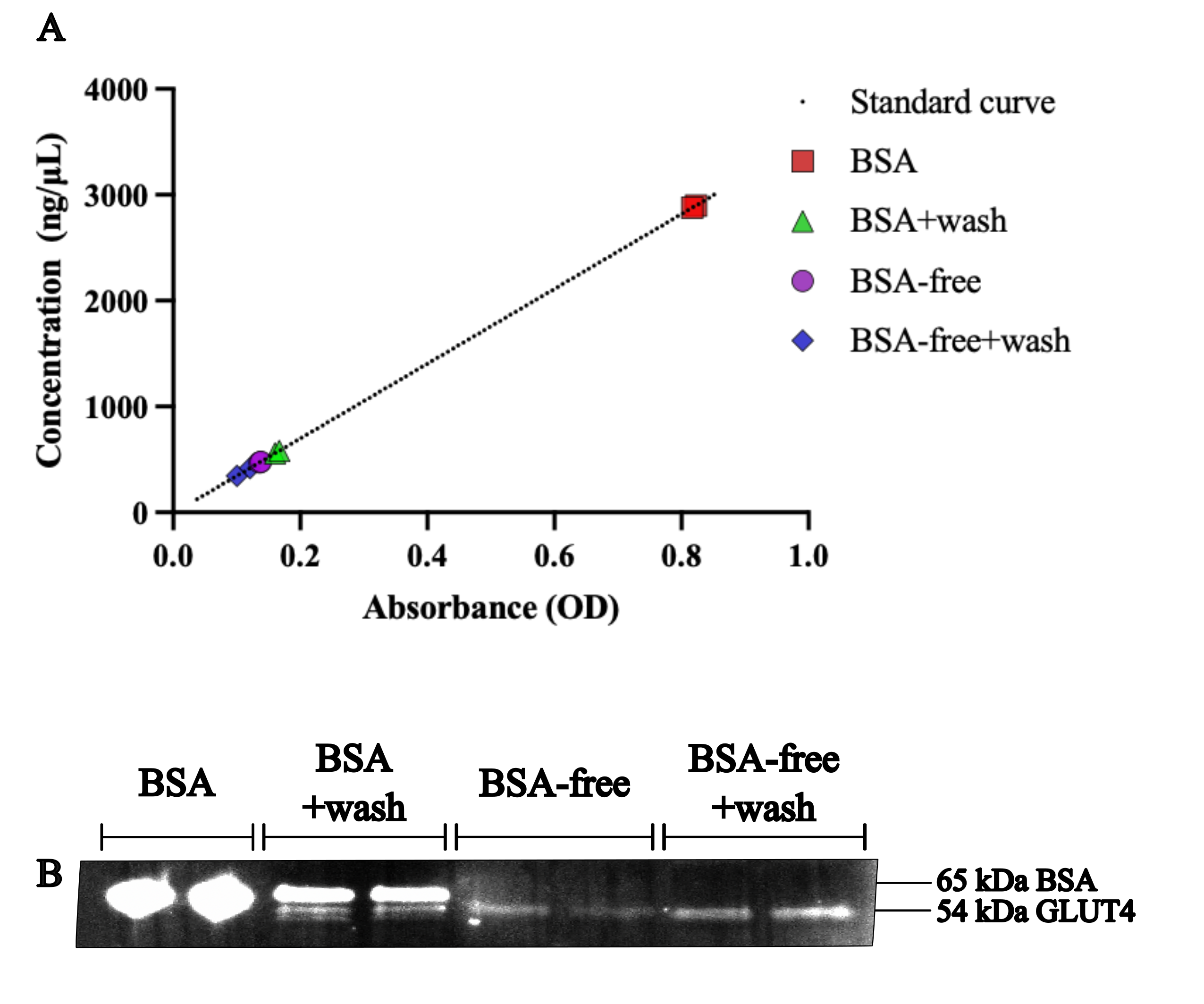

Supplement: S3 Fig — (A) Protein concentrations, determined with a BSA standard curve and absorption measurement using a spectrophotometer with duplicates for all conditions. (B) GLUT4 stain, shown with increased exposure to better demonstrate specific GLUT4 staining in the BSA free samples. (TIF) [file pone.0328136.s007.tif]

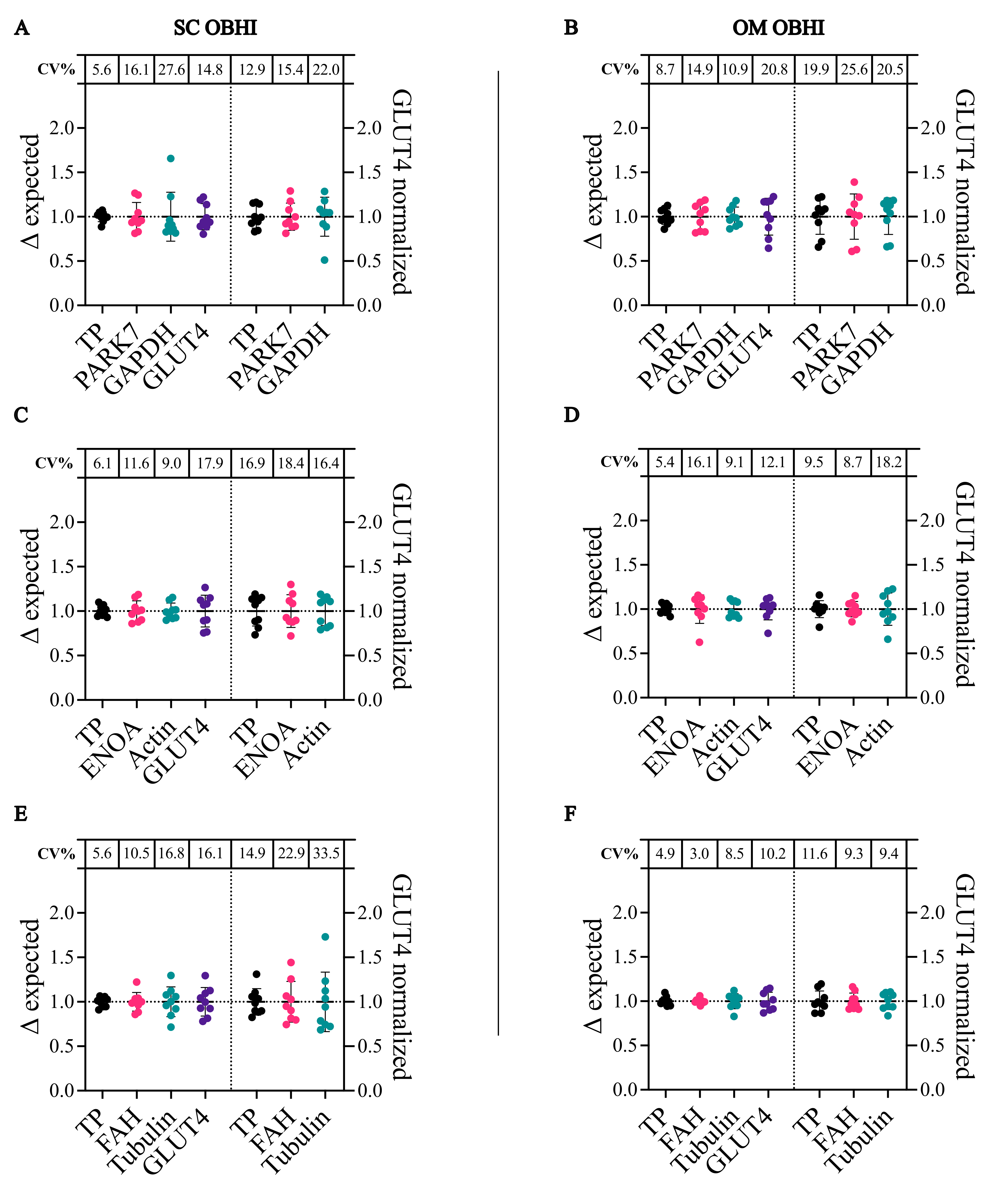

Supplement: S4 Fig — Coefficient of variation (CV) for total protein (TP) and respective housekeeping proteins, normalized to the sum (left panels), and for GLUT4 normalized to TP or housekeeping proteins (right panels). Each dot represents one well – a technical replicate of the same lysate sample run in separate lanes. Protein isolated from subcutaneous (SC) or omental (OM) adipocytes. Total protein demonstrated the lowest CV across all technical replicate blots, except when compared to FAH in the OM sample (A–F). When normalizing GLUT4, total protein showed the lowest CV in three blots (A, B and E), while actin (C), ENOA (D) and FAH and tubulin (F) exhibited lower CVs in three blots. (TIF) [file pone.0328136.s008.tif]

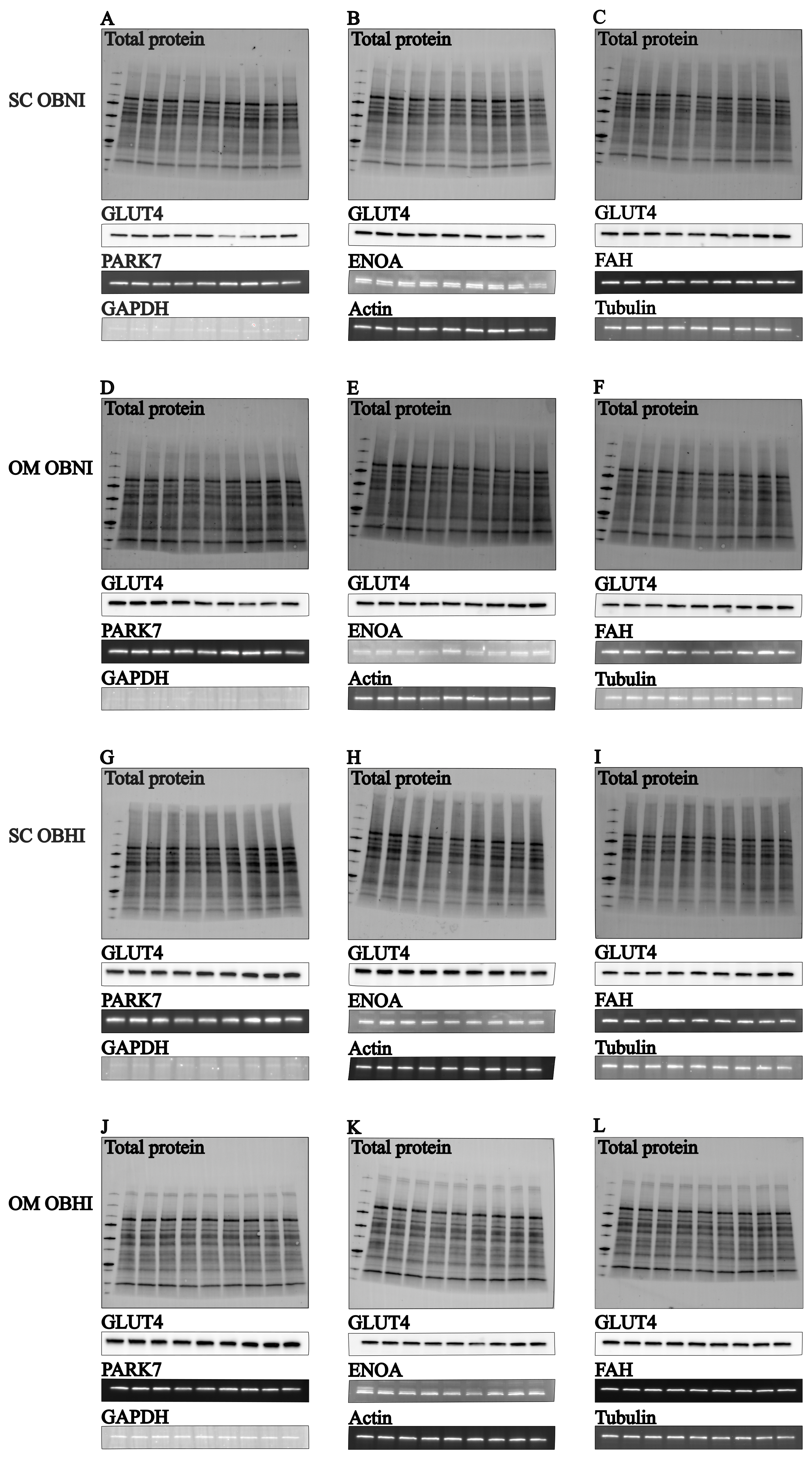

Supplement: S5 Fig — Technical replicate blots from Fig 2 and S3 Fig. (A) Subcutaneous obese normoinsulinemic (SC OBNI), total protein (TP), PARK7, GAPDH and GLUT4, (B) SC OBNI, TP, ENOA, actin and GLUT4, (C) SC OBNI, TP, FAH, tubulin and GLUT4, (D) omental (OM) OBNI, TP, PARK7, GAPDH and GLUT4, (E) OM OBNI, TP, ENOA, actin and GLUT4, (F) OM OBNI, TP, FAH, tubulin and GLUT4. Technical replicate blots from S3 Fig: (G) Subcutaneous obese hyperinsulinemic (SC OBHI), TP, PARK7, GAPDH and GLUT4, (H) SC OBHI, TP, ENOA, actin and GLUT4, (I) SC OBHI, TP, FAH, tubulin and GLUT4, (J) OM OBHI, TP, PARK7, GAPDH and GLUT4, (K) OM OBHI, TP, ENOA, actin and GLUT4, (L) OM OBHI, TP, FAH, tubulin and GLUT4. (TIF) [file pone.0328136.s009.tif]

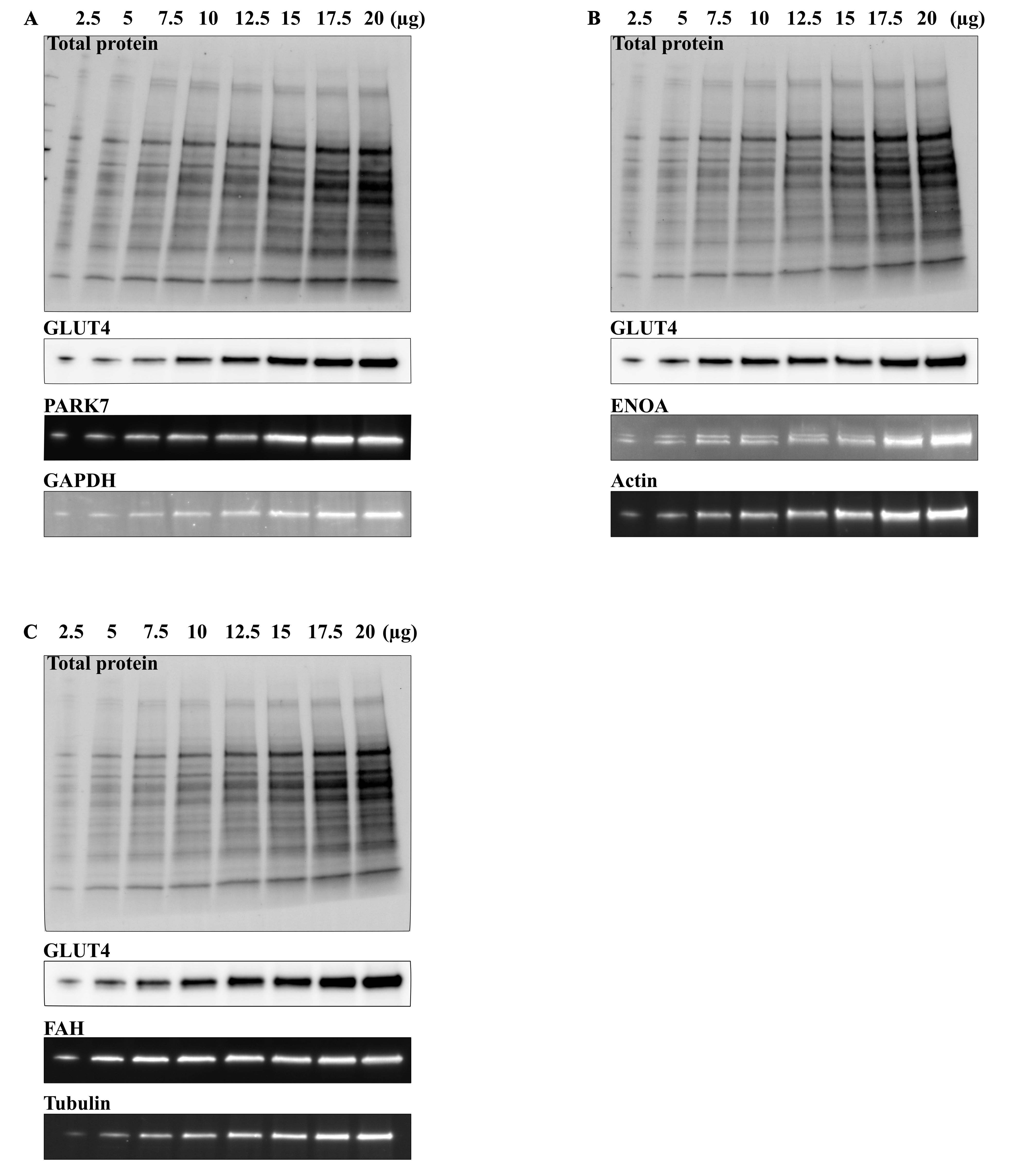

Supplement: S6 Fig — omental obese hyperinsulinemic (OM OBHI) lysate loaded on a gradient in 2.5 µg increments (2.5–20 µg). (A) Stain-free image of total protein (TP) and proteins stains for GLUT4, PARK7 and GAPDH, (B) GLUT4, ENOA and Actin, (C) GLUT4, FAH and tubulin. (TIF) [file pone.0328136.s010.tif]

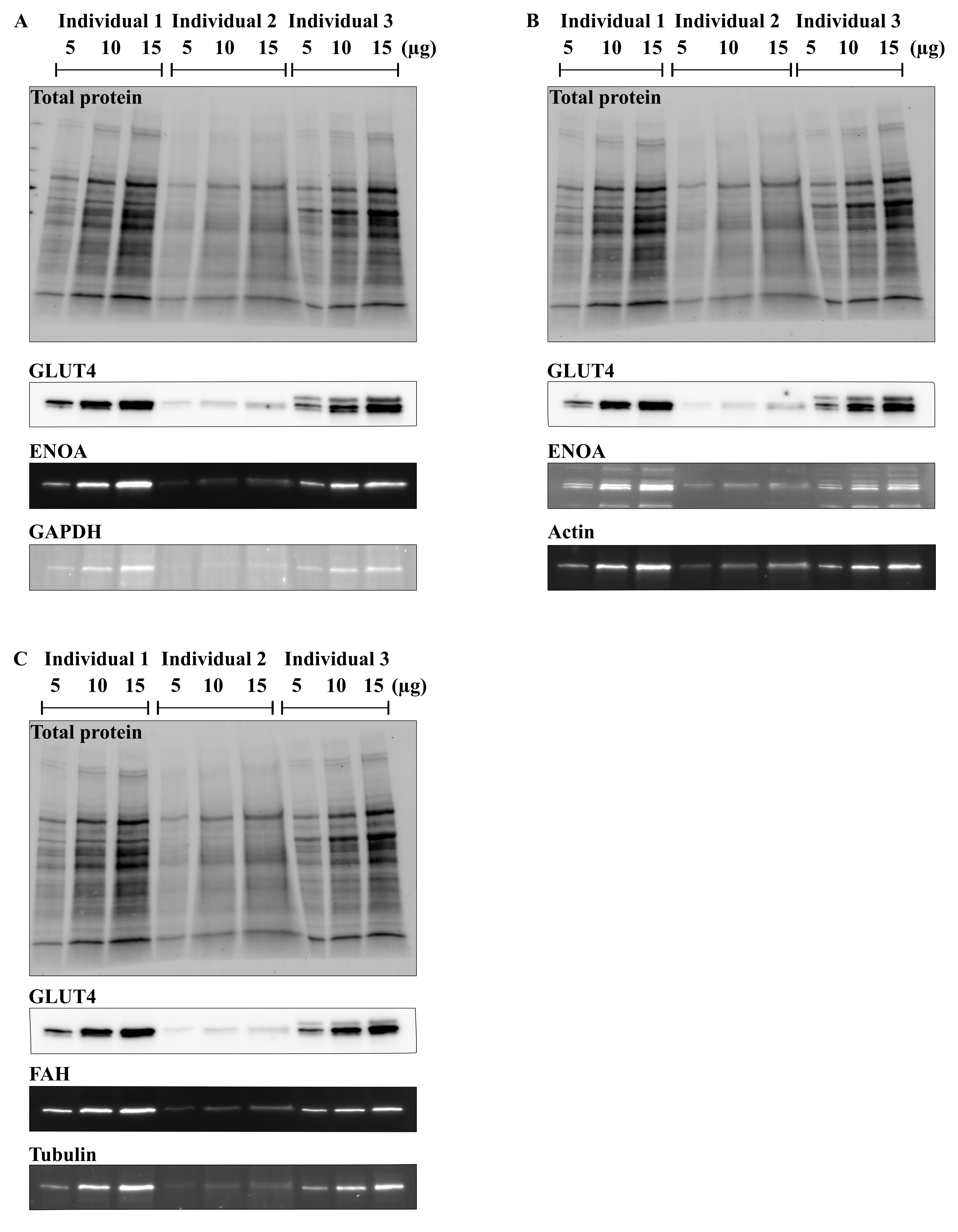

Supplement: S7 Fig — (A) Stain-free image of total protein (TP) and GLUT4, PARK7 and GAPDH staining. (B) Stain-free image of TP and GLUT4, ENOA and actin staining. (C) Stain-free image of TP and GLUT4, FAH and tubulin staining. (TIF) [file pone.0328136.s011.tif]
