# Supplementary material for: Superior normalization using total protein for western blot analysis of human adipocytes
Source: PLoS One. 2025 Jul 22;20(7):e0328136. doi: 10.1371/journal.pone.0328136 (PMC12282925; doi:10.1371/journal.pone.0328136)

Blot figure 1 BSA contamination + Blots s figure 2 BSA contamination

Total protein

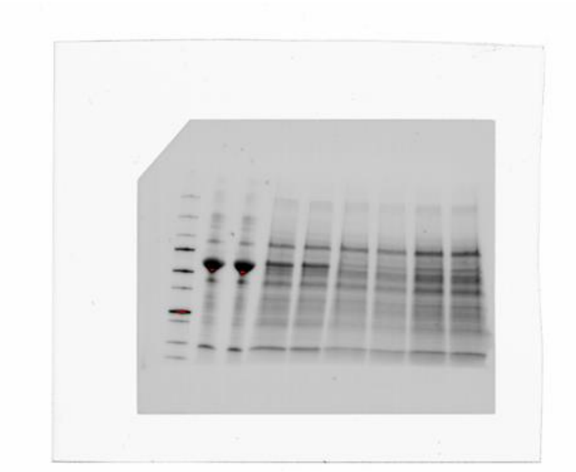

GLUT4

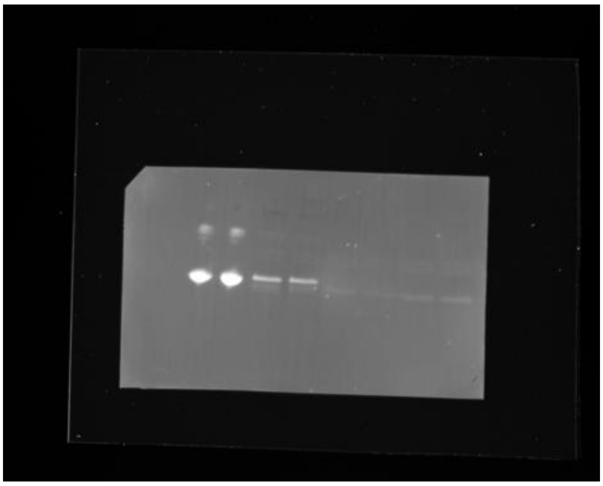

Tubulin

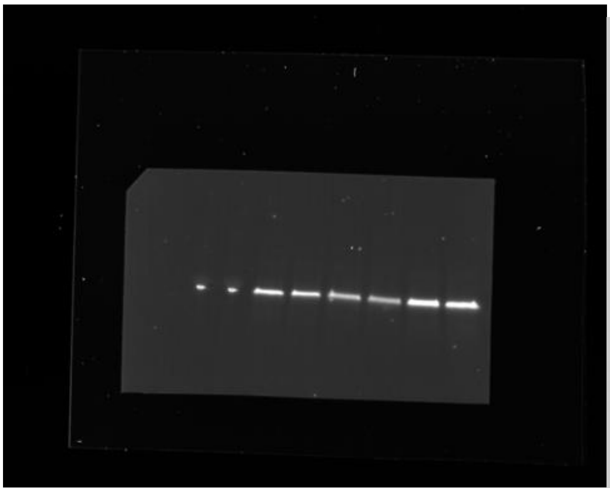

BSA

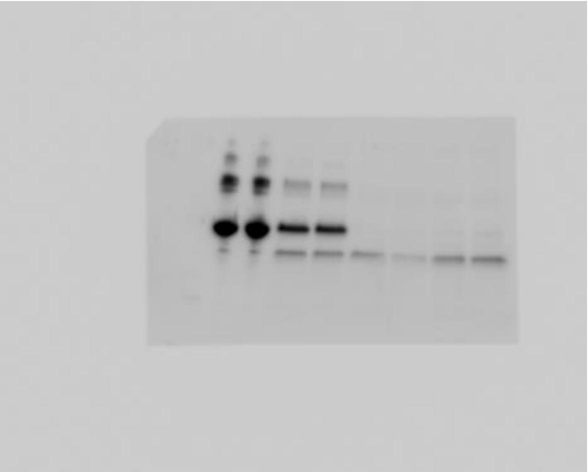

Blots figure 2 technical replicates SC and OM OBNI

OBNI SC

Blot 1

GAPDH

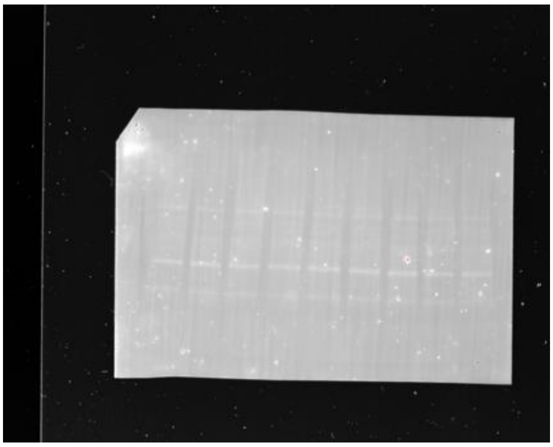

Total protein

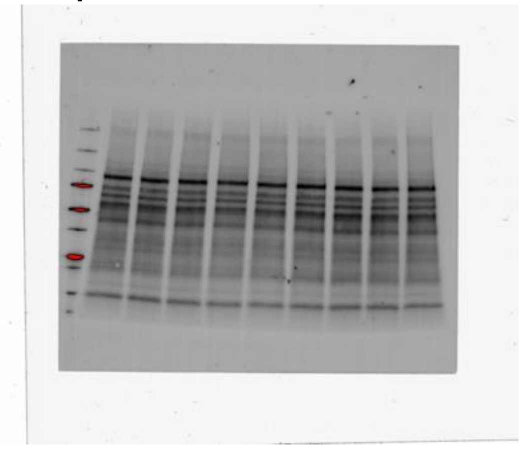

PARK7

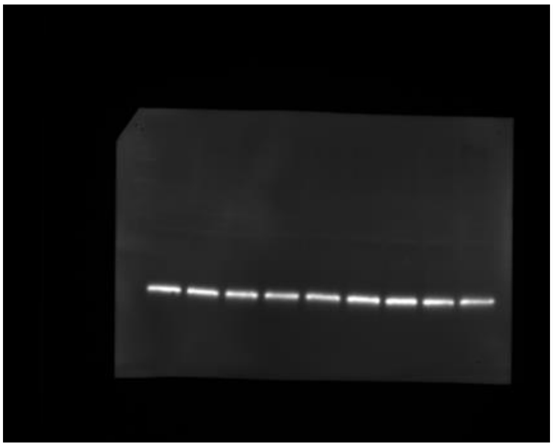

GLUT4

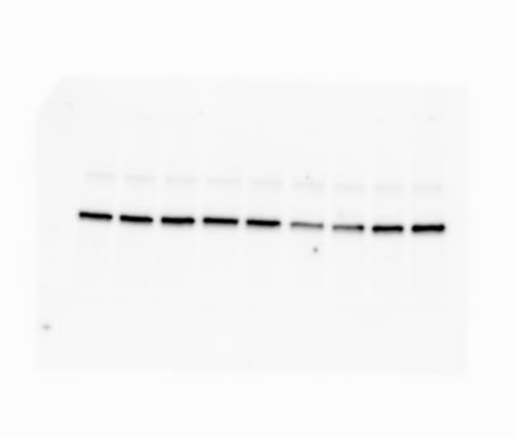

Blot 2

Total protein

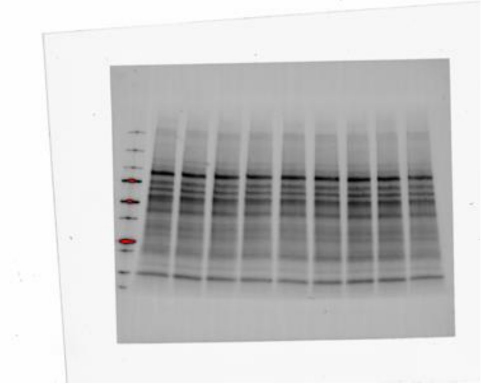

GLUT4

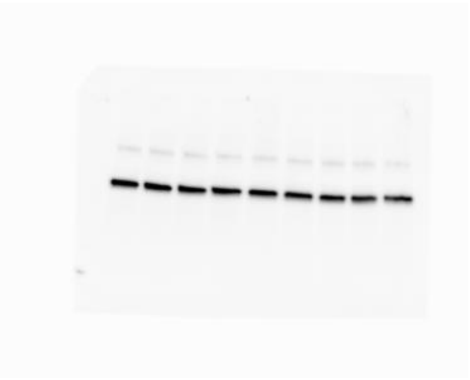

ENOA

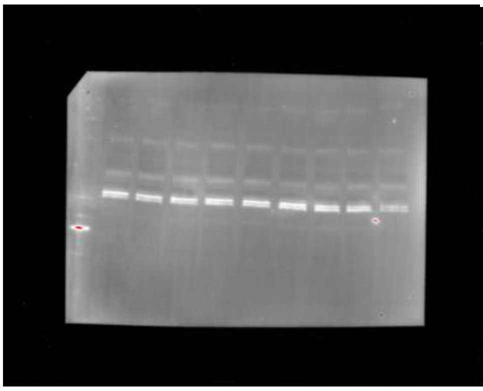

Actin

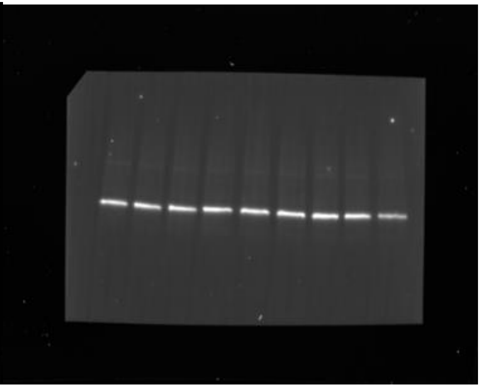

Blot 3

Total protein

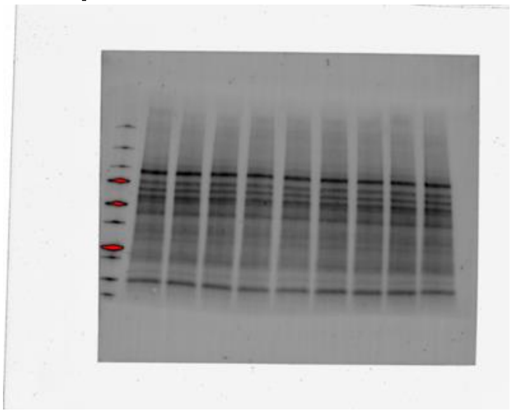

GLUT4

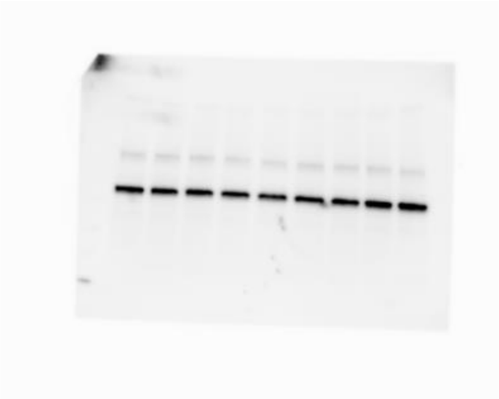

FAH

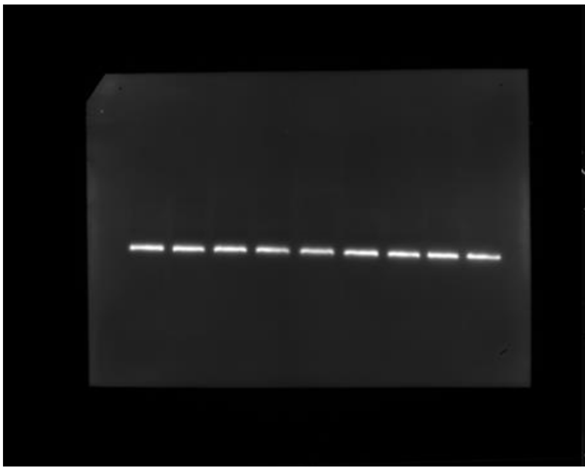

Tubulin

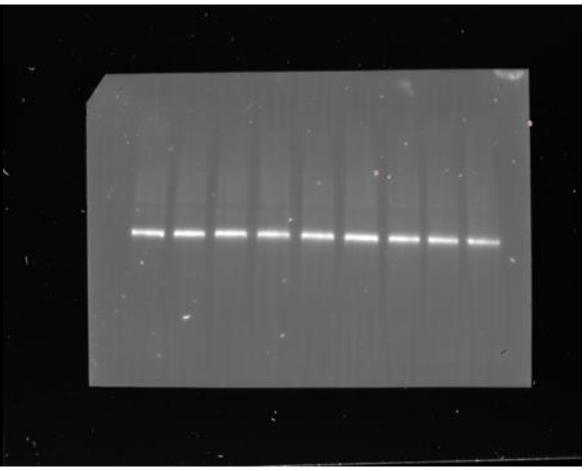

OBNI OM

Blot 1

Total protein

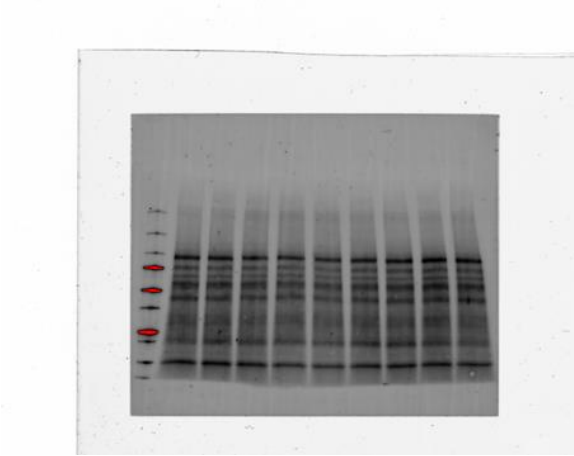

GLUT4

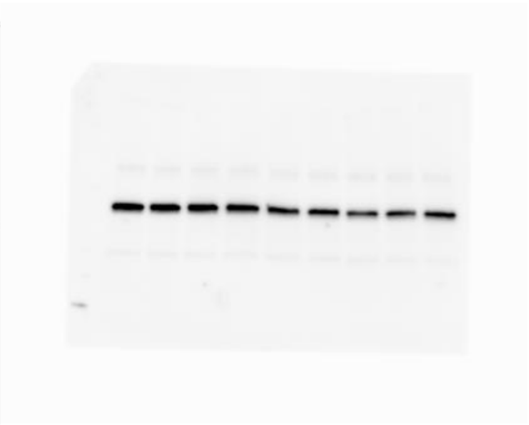

PARK7

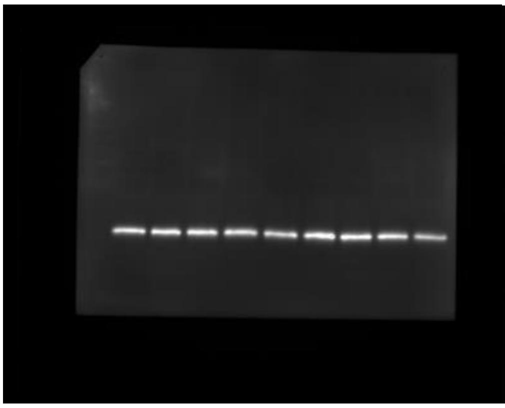

GAPDH

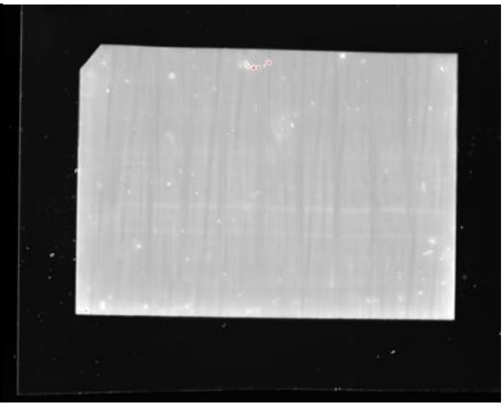

Blot 2

Total protein

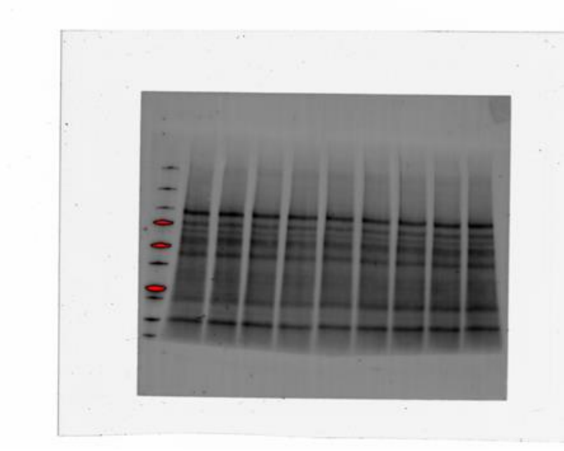

GLUT4

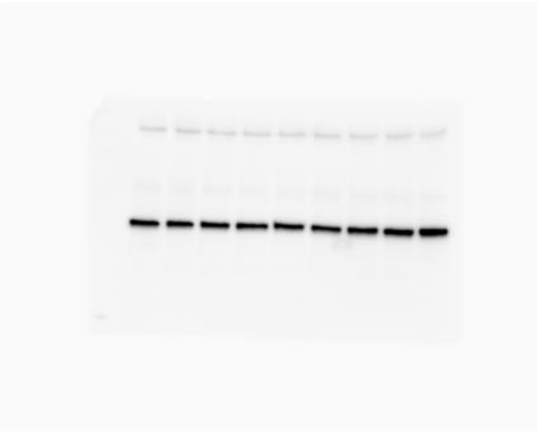

ENOA

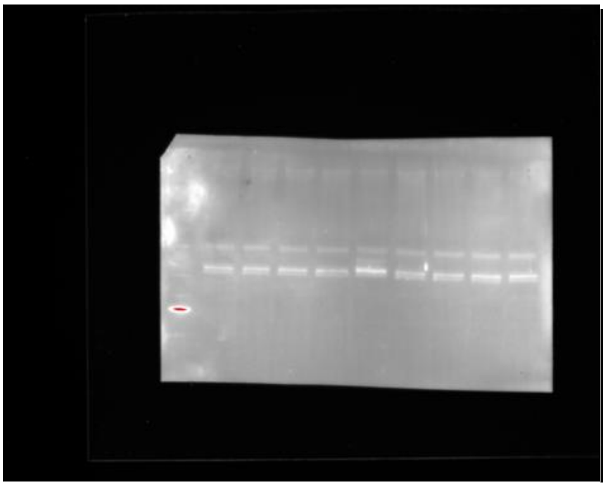

Actin

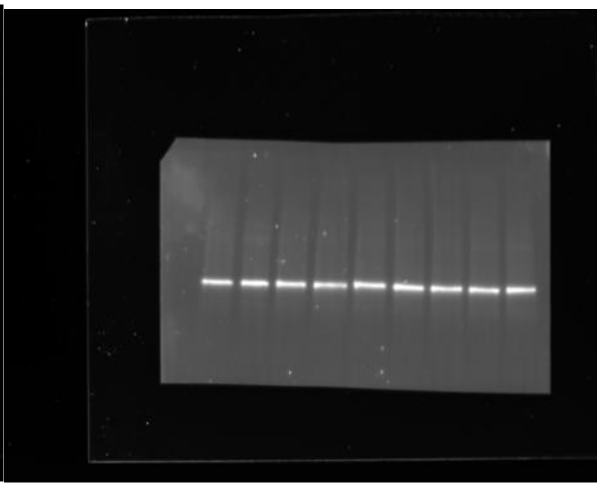

Blot 3

Total protein

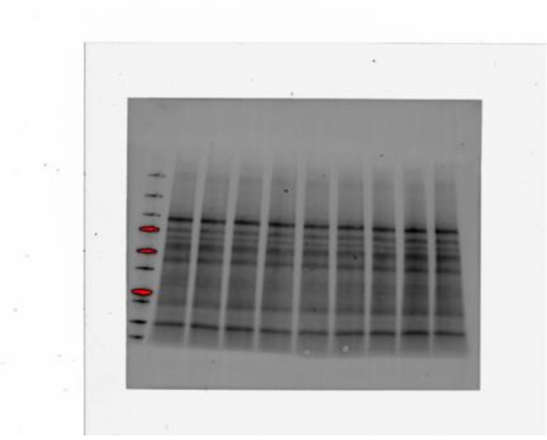

GLUT4

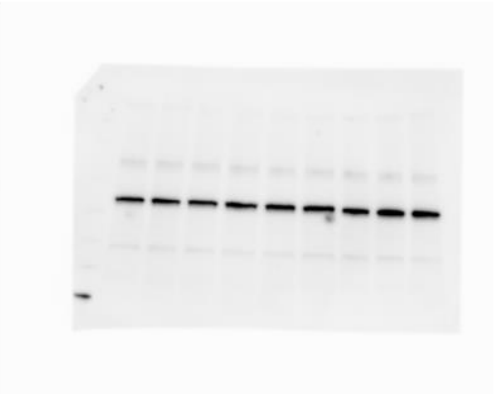

FAH

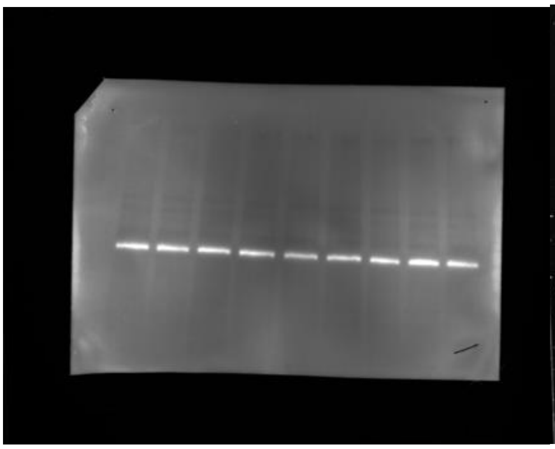

Tubulin

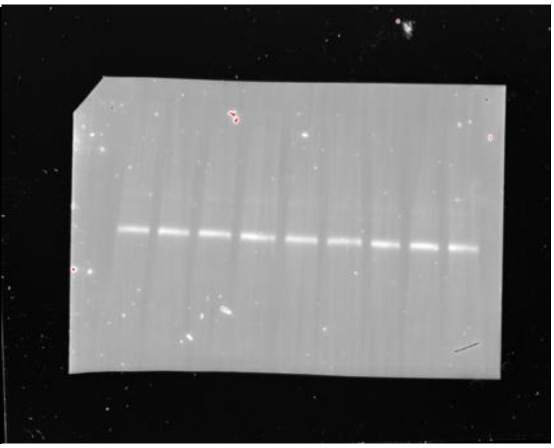

Blots figure 3 Dynamic range, loaded on a protein gradient from 2.5-20µg in 2.5µg increments.

Blot 1

Total protein

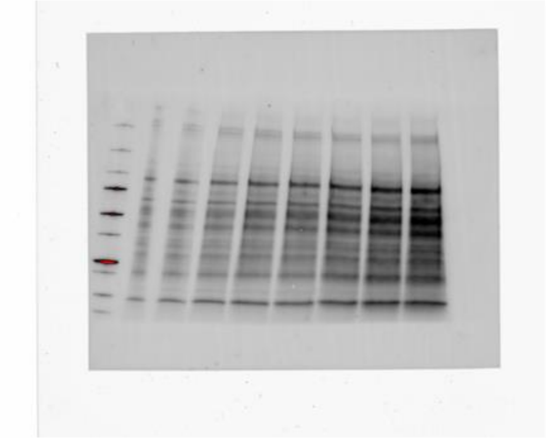

GLUT4

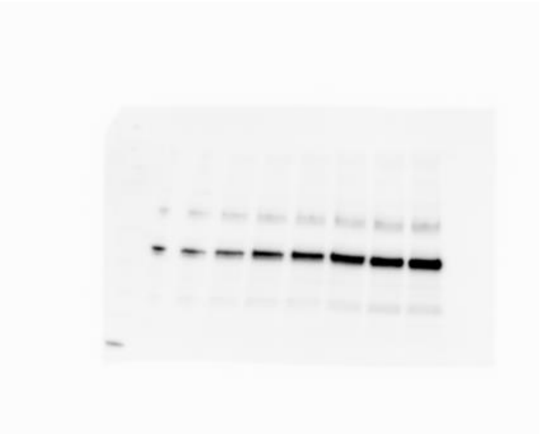

PARK7

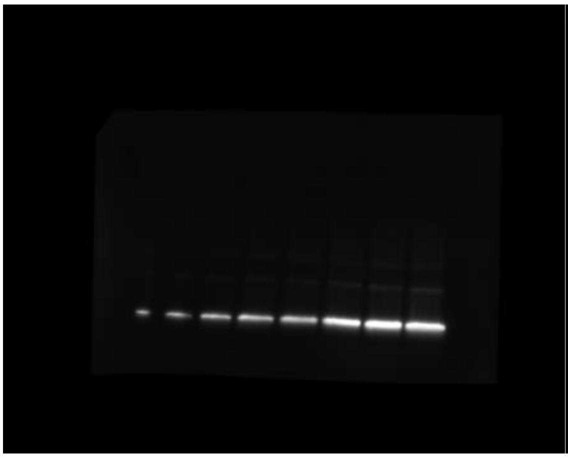

GAPDH

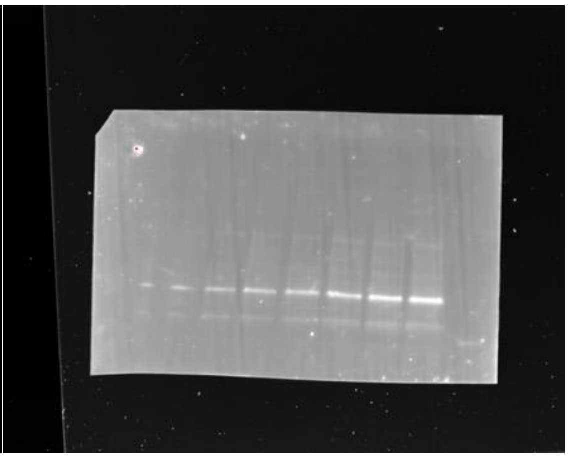

Blot 2

Total protein

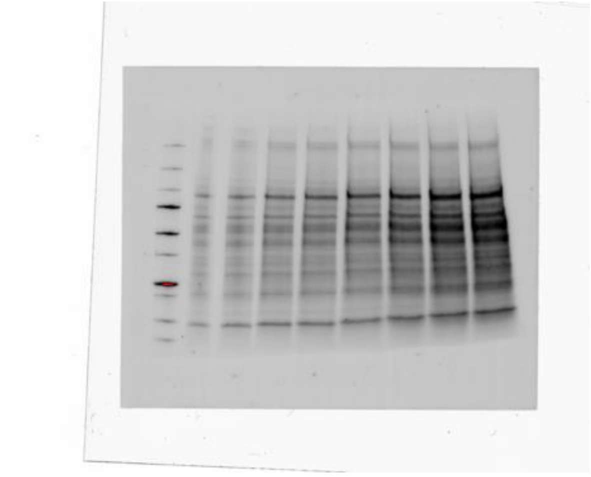

GLUT4

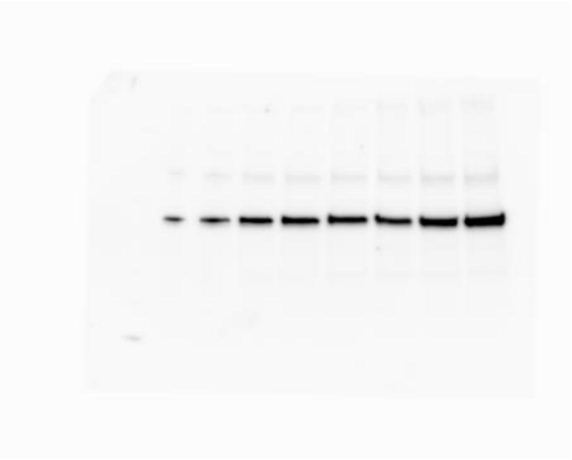

ENOA

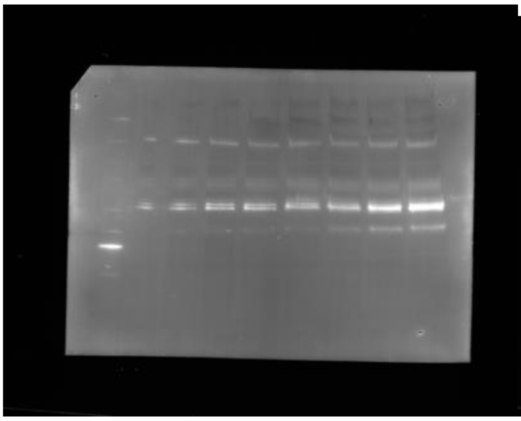

Actin

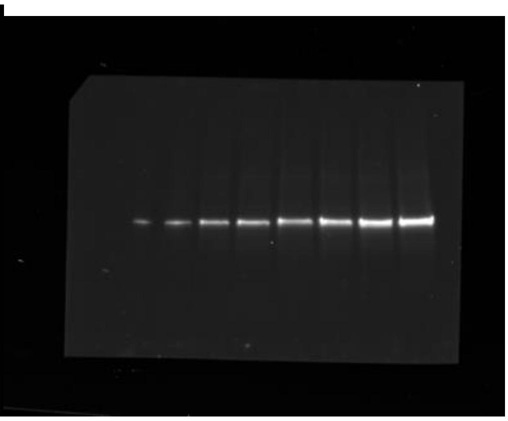

Blot 3

Total protein

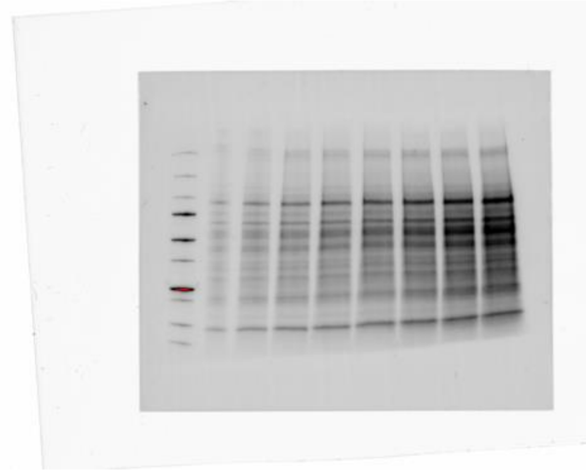

GLUT4

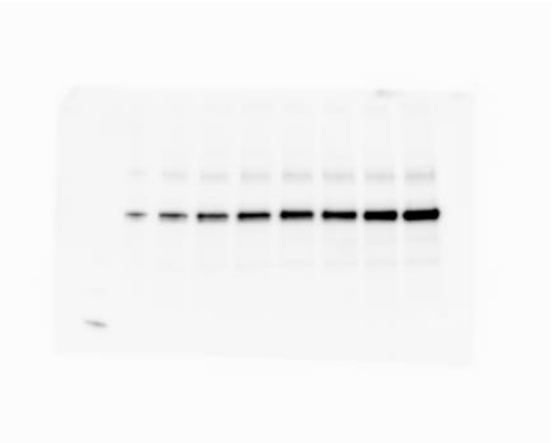

FAH

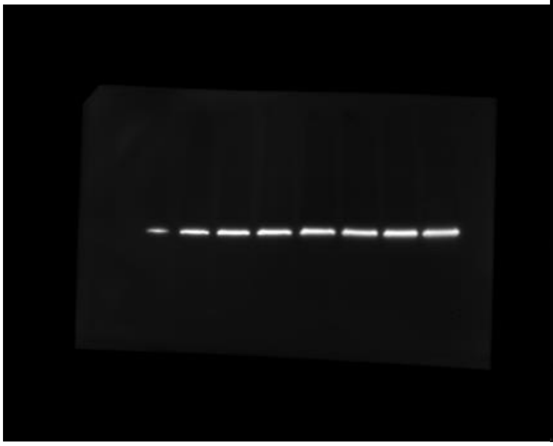

Tubulin

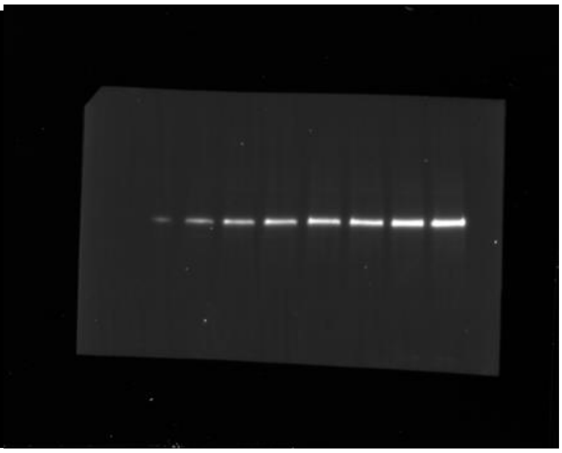

Blots figure 4. Three different individuals loaded at 5, 10 and 15 µg

Blot 1

Total protein

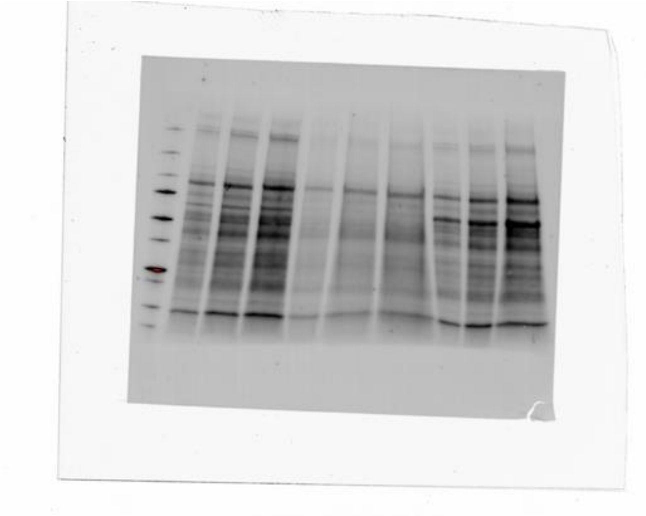

GLUT4

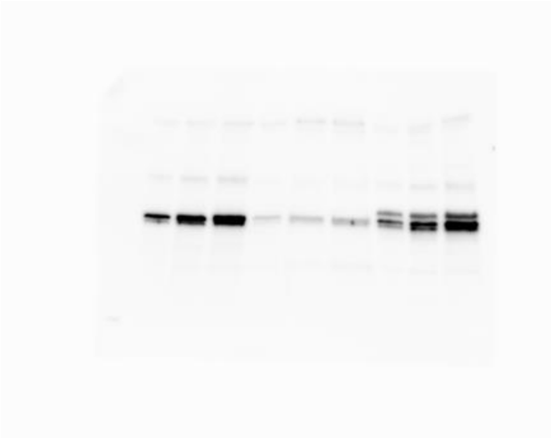

PARK7

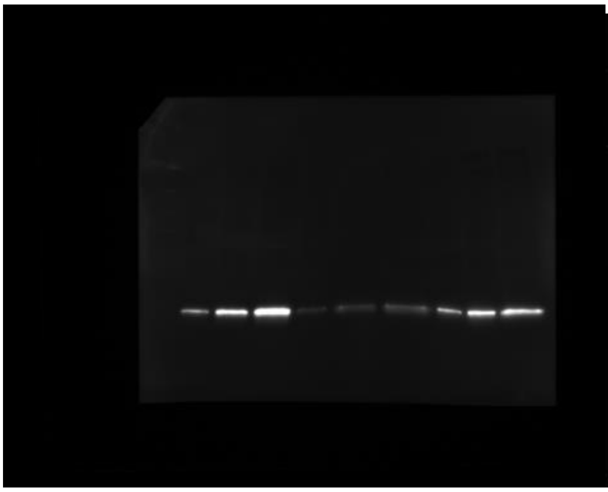

GAPDH

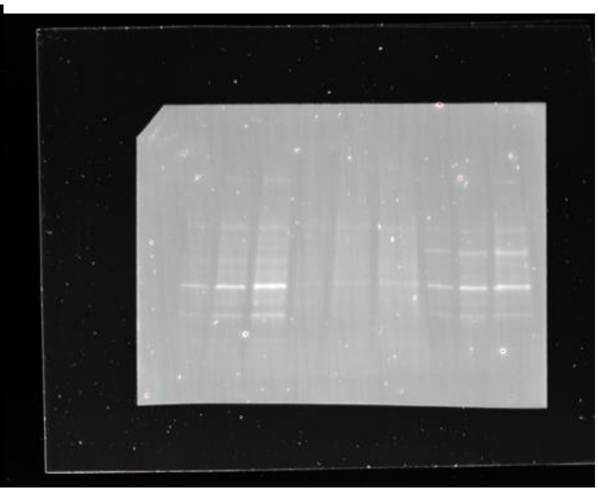

Blot 2

Total protein

GLUT4

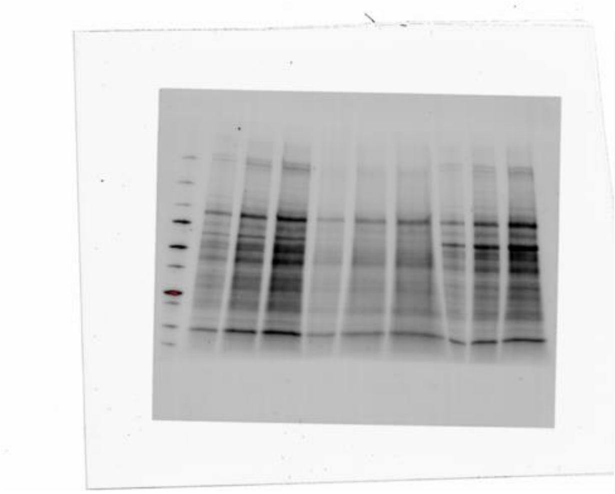

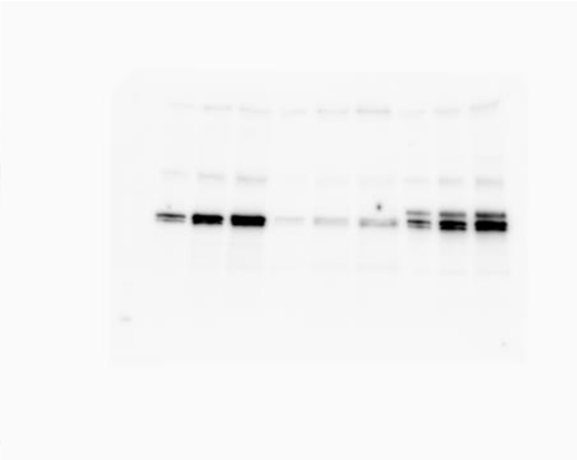

ENOA

Actin

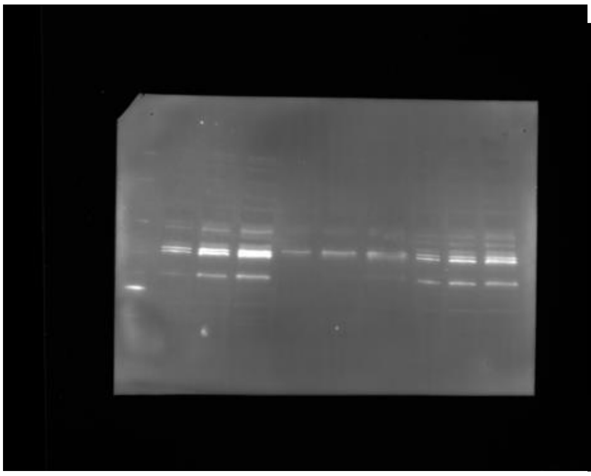

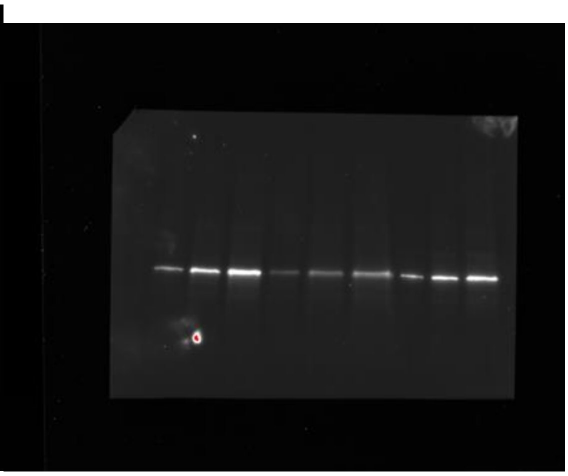

Blot 3

Total protein

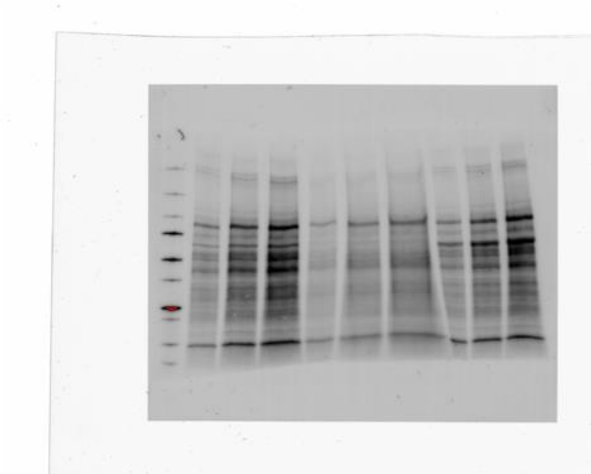

GLUT4

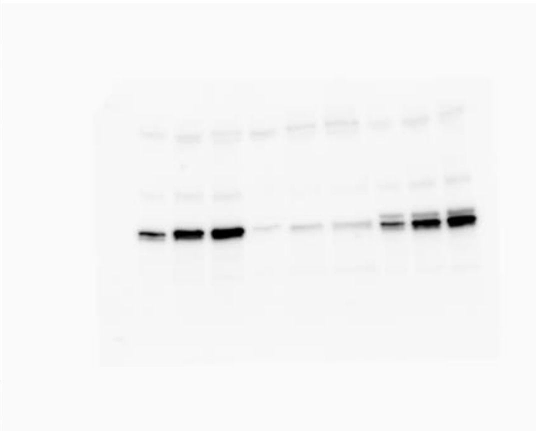

FAH

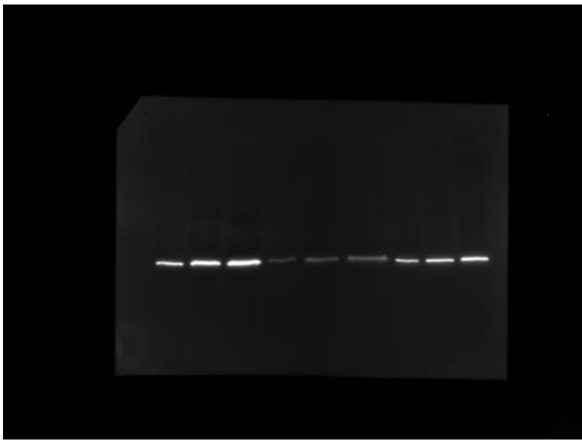

Tubulin

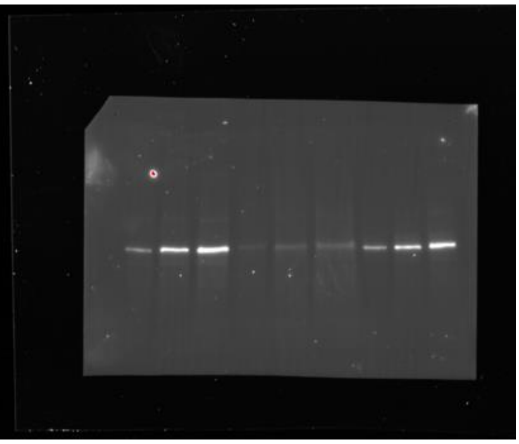

Blots s figure 3 Technical replicates SC and OM HI

OBHI SC

Blot 1

Total protein

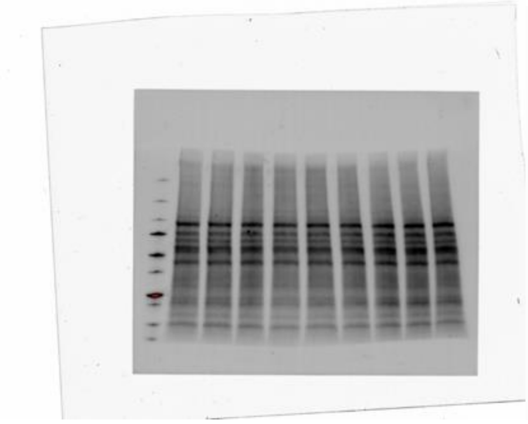

GLUT4

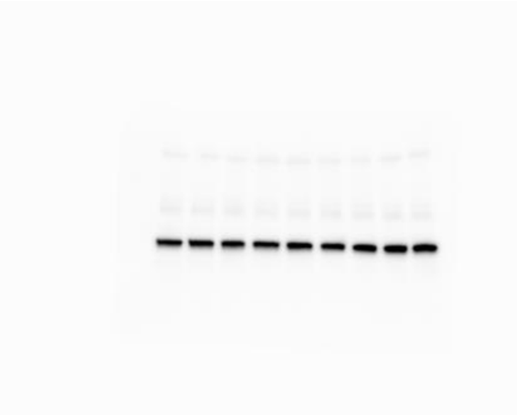

PARK7

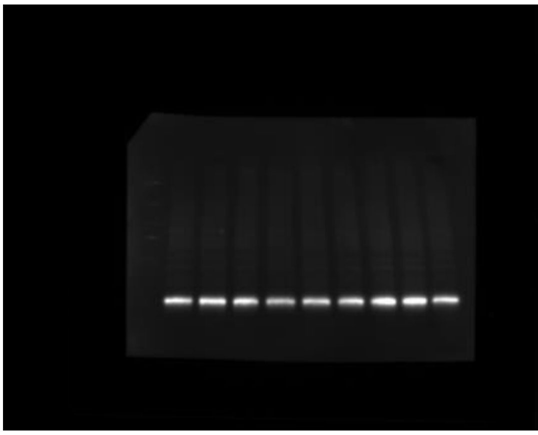

GAPDH

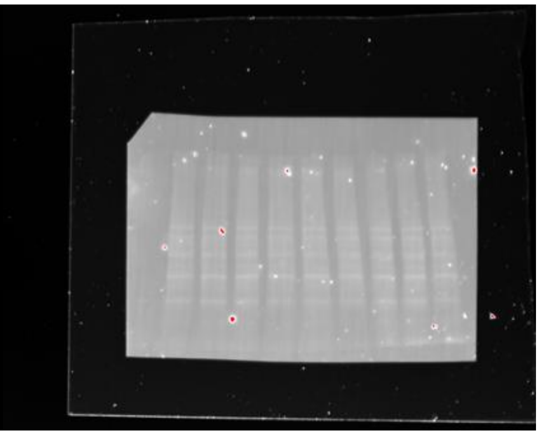

Blot 2

Total protein

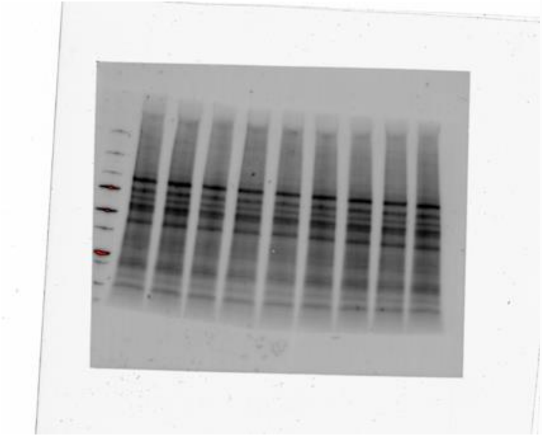

GLUT4

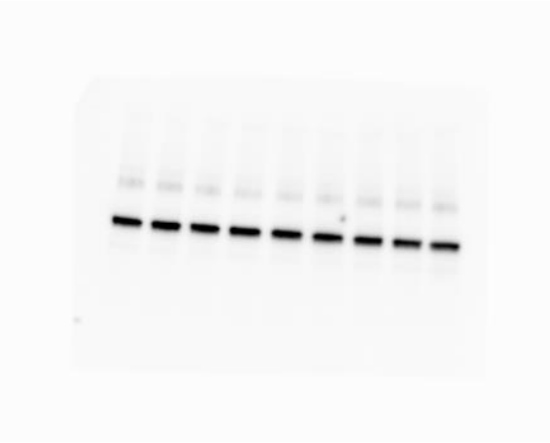

ENOA

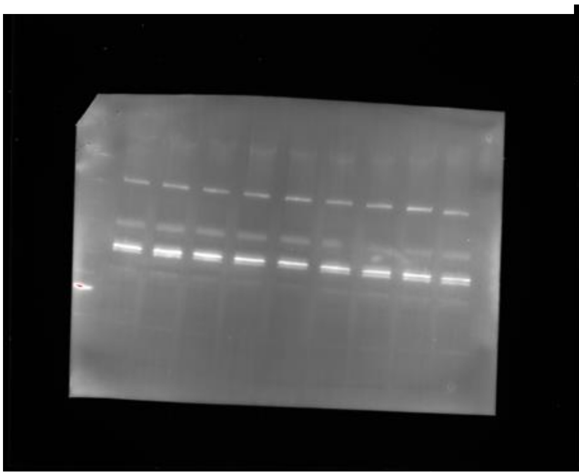

Actin

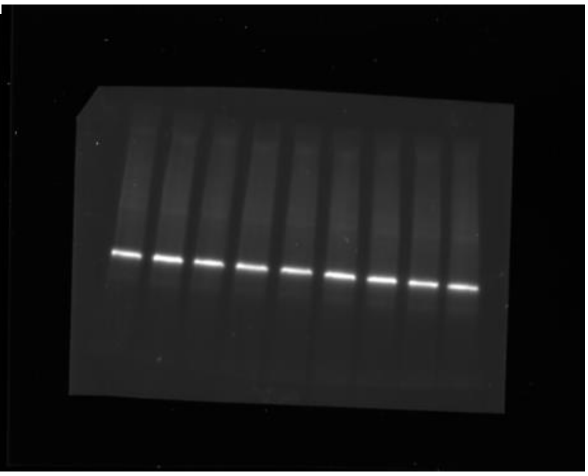

Blot 3

Total protein

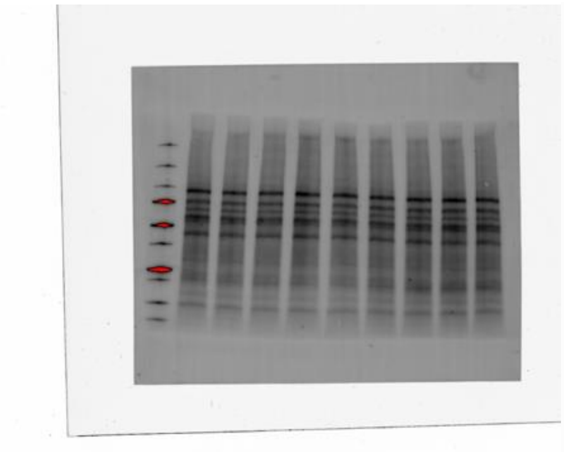

GLUT4

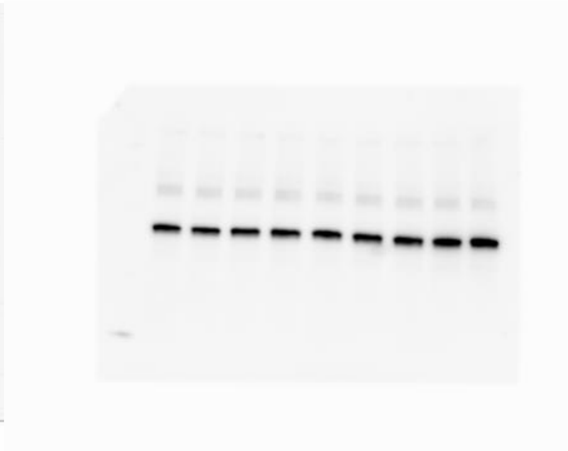

Tubulin

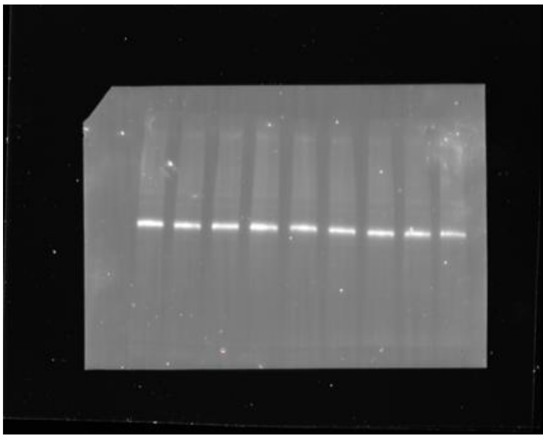

FAH

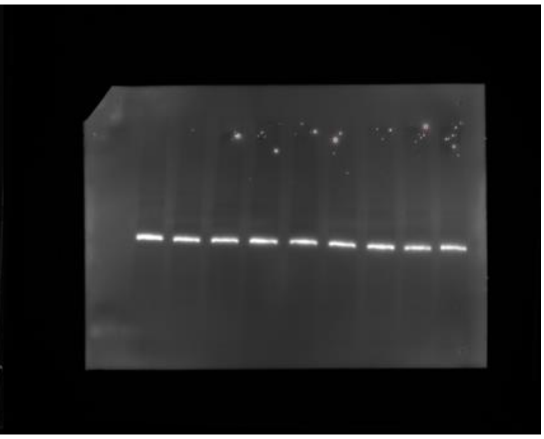

OBHI OM

Blot 1

Total protein

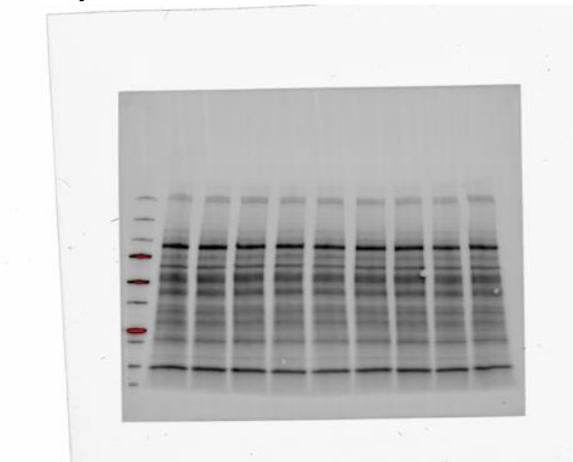

GLUT4

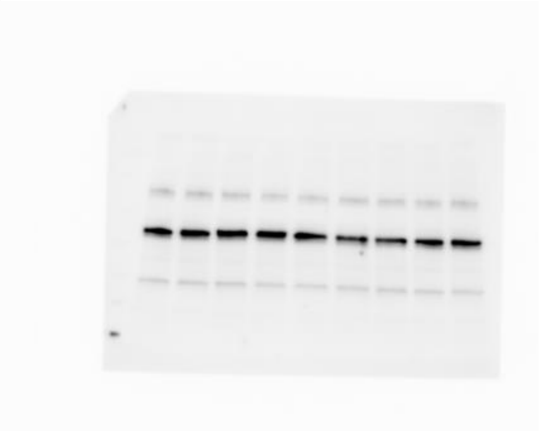

PARK7

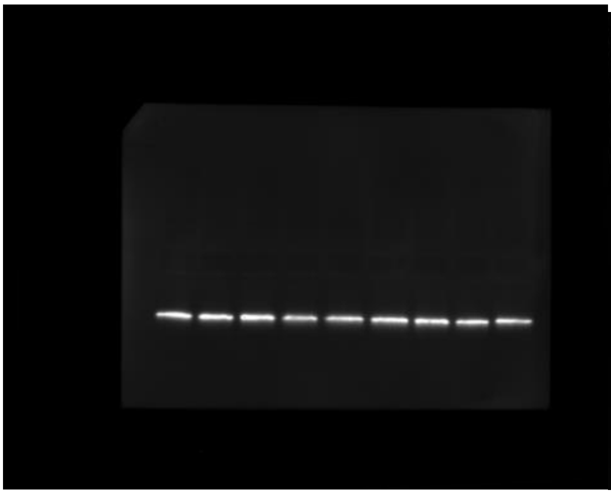

GAPDH

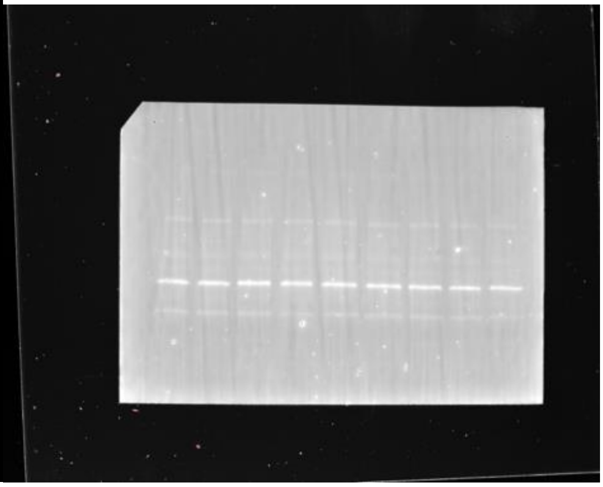

Blot 2

Total protein

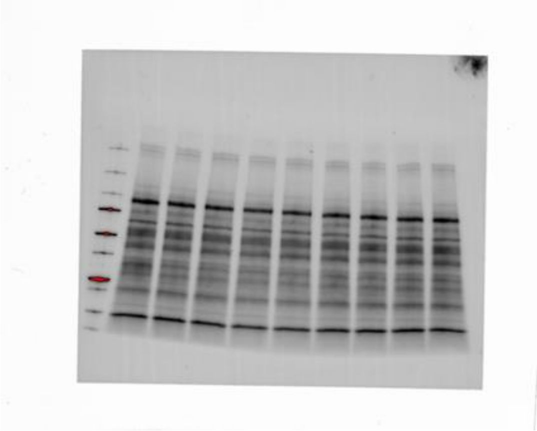

GLUT4

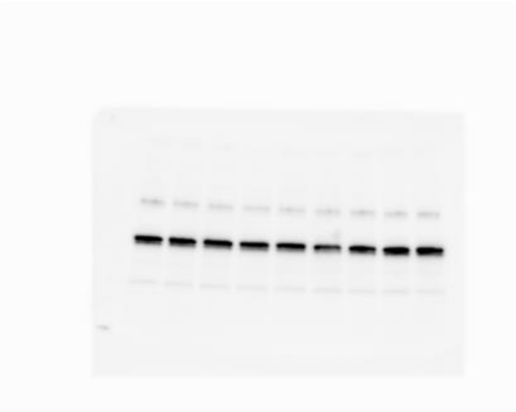

ENOA

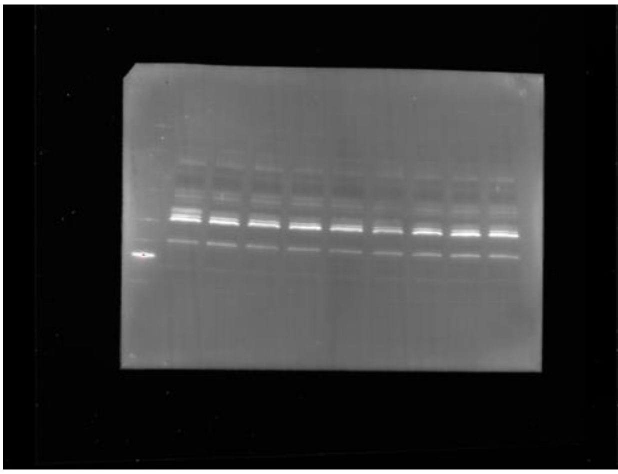

Actin

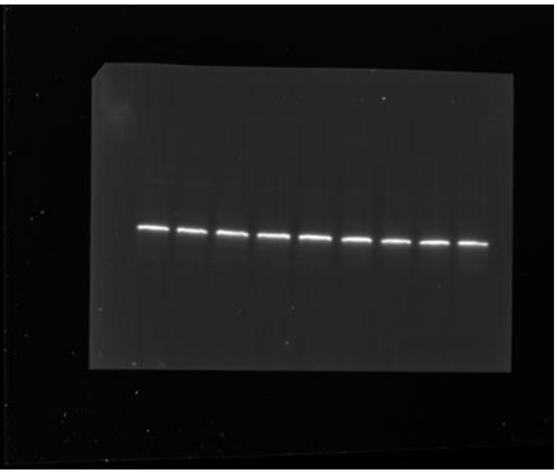

Blot 3

Total protein

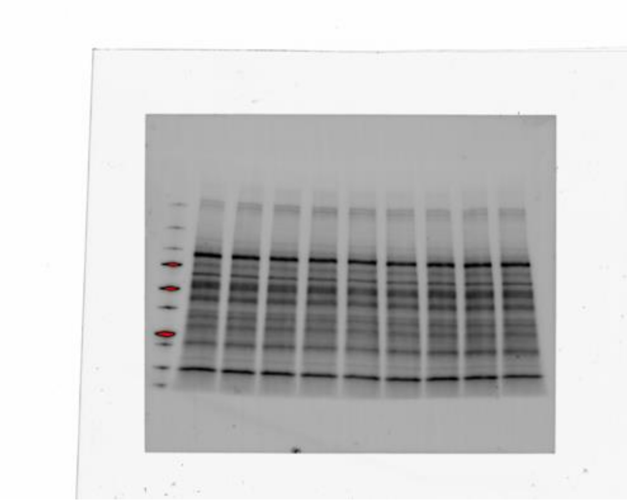

GLUT4

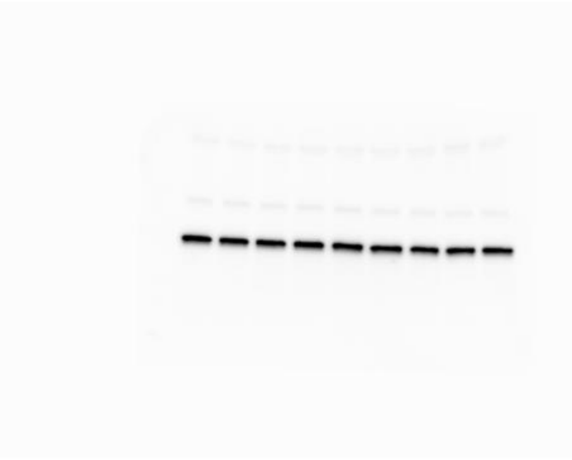

Tubulin

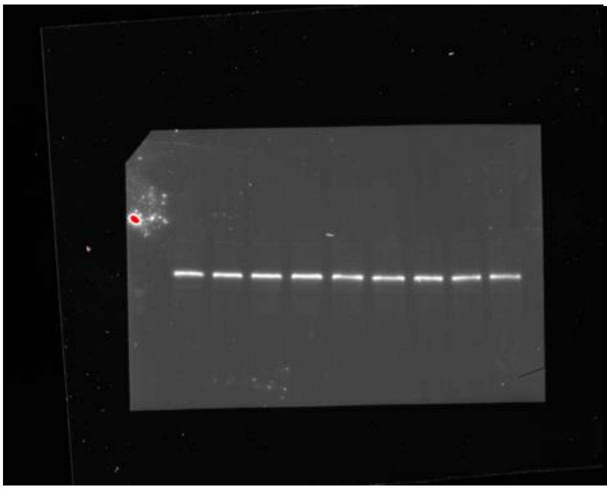

FAH

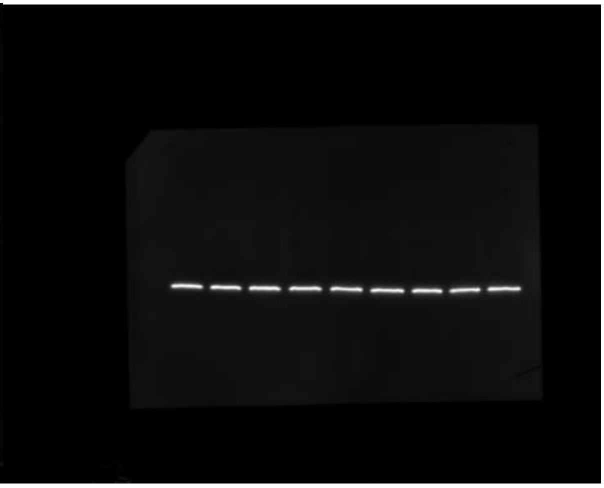

Supplement: S1 Raw images — (PDF) [file pone.0328136.s012.pdf]
