# Supplementary material for: Superior normalization using total protein for western blot analysis of human adipocytes
Source: PLoS One. 2025 Jul 22;20(7):e0328136. doi: 10.1371/journal.pone.0328136 (PMC12282925; doi:10.1371/journal.pone.0328136)
Supplement: Attachment — Submitted filename: Response to reviewers.pdf [file pone.0328136.s014.pdf]

We are grateful to the reviewers for their thoughtful and constructive feedback. We have put considerable effort into addressing all comments, and the revision process has helped us create a more comprehensive and user-friendly manuscript.

Individual responses to each review are below. Reviewer comments are in black, author responses in blue.

Key improvements include:

1. Detailed guidance on normalization method selection
2. Expanded statistical methodology and referencing
3. Thorough discussion of technical considerations such as BSA handling and tryptophan metabolism
4. Enhanced clarity throughout the figures and text

We believe these changes have substantially strengthened the utility of our work, making it a valuable resource for the research community.

Reviewer 1

1. *"The introduction effectively sets up the study's objective, emphasizing the need for improved normalization strategies in Western blot analysis."*

We thank the reviewer for their positive feedback on our manuscript.

2. *"Consider briefly addressing why TP normalization has not yet become a universal standard, despite its advantages, to contextualize the significance of this research."*

We appreciate this suggestion and have added a new paragraph to the introduction addressing these important points:

Despite these established advantages, TP normalization has not yet achieved universal adoption in western blot analysis. The persistence of traditional housekeeping protein methods can be attributed to several factors, i) historical precedent has maintained housekeeping proteins as the default choice in many laboratories, particularly given their extensive representation in published literature, ii) early iterations of TP detection methods introduced technical complexities and potential interference with subsequent immunodetection steps and iii) the requirement for specialized imaging equipment and standardized detection protocols has posed implementation barriers. Moreover, the diverse array of TP detection methods, from classical chromogenic stains to stain-free technologies, has limited the establishment of a unified normalization protocol.

3. *"The methods section is comprehensive, but some procedural descriptions, such as the justification for excluding BSA in adipocyte isolation, could benefit from further elaboration."*

We thank the reviewer for this insightful comment. As detailed in the results section and illustrated in Figure 1, we identified BSA as a protein contaminant during adipocyte isolation and demonstrated its impact on western blot results. In response to the reviewer's suggestion, we have expanded the discussion to provide additional justification for the exclusion of BSA in adipocyte isolation protocols. This additional text can be found in the discussion.

Although tubulin outperformed TP in the presence of BSA, the fundamental issues with using housekeeping proteins as normalization references, highlighted in this study, remain. For optimal western blot normalization, it is preferable to eliminate exogenous protein contamination, such as BSA, rather than relying on housekeeping proteins as an alternative. In our experiments, excluding BSA from the adipocyte isolation protocol had no effect on adipocyte yield, however validation of removing BSA for downstream applications is needed. For experiments where the use of BSA is unavoidable, actin, as identified in this study, may

serve as a more suitable normalization reference. However, whenever possible, adopting BSA-free protocols is strongly recommended to fully leverage the superior normalization capabilities of TP.

4. *"In the protein extraction and quantification section, it might help to clarify why the BCA assay was chosen over other protein quantification methods."*

We thank the reviewer for this important methodological clarification. The choice of BCA over a Bradford assay was based on several experimental considerations. First, our optimized protein extraction protocol utilizes 1% SDS, which we determined to be optimal for efficient extraction of typical transmembrane protein (insulin receptor B, INSR-B) and nuclear proteins (APE2) compared to other lysis buffers including RIPA (see figure below). While SDS effectively solubilizes proteins across cellular compartments, it is known to interfere with Bradford reagents, generating artificially high readings even in blank samples (see figure below). The BCA assay, in contrast, maintains compatibility with the SDS concentrations we use, and provides reliable protein quantifications in our experimental conditions. We validated this experimentally by comparing Bradford and BCA assay quantification using our extraction protocol and loading the same sample for both assays (Figures and data tables are shown below, for reviewer only).

An isolated adipocyte sample was divided into equal volumes and processed with the indicated lysis buffers. Analysis showed that SDS-based extraction yielded substantially higher protein concentrations compared to alternative buffers. In a separate analysis of these protein isolates (Reviewer 1, Figure 1), SDS buffer produced the strongest signal for the transmembrane protein insulin receptor (beta domain). When normalizing to TP, SDS showed the strongest signal amongst all buffers.

Lysis	Conc µg/µl	Volume
**1% SDS**	0,59	500 µL
**M-PER**	0,209	500 µL
**RIPA**	0,29	500 µL

Reviewer 1, Table 1. Protein yield from the same sample using different lysis buffers for protein isolation.

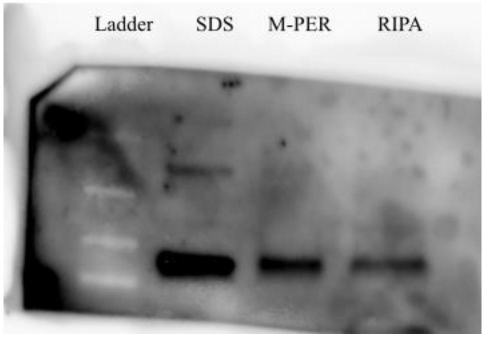

Reviewer 1, Figure 1. Western blot of INSR B staining from lysates obtained using different lysis buffers on the same sample.

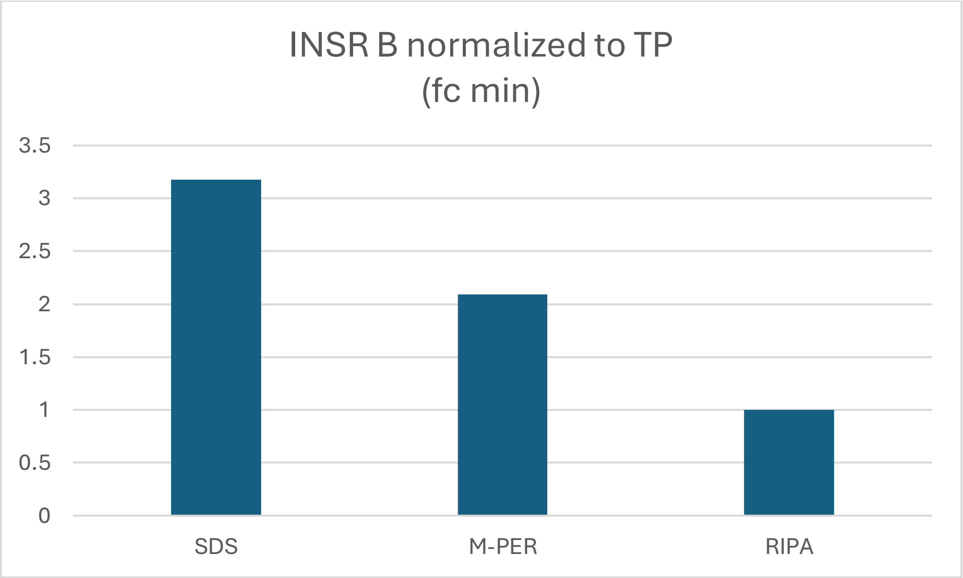

Reviewer 1, Figure 2. TP normalized values from western blot of insulin receptor B. Results demonstrate variations in the detection of insulin receptor B depending on the lysis buffer used, emphasizing the impact of buffer choice on protein extraction and subsequent analysis.

To compare protein quantification methods, three patient protein lysates were analyzed alongside a standard curve, lysis buffer (SDS) blank, and H_2O control using both Bradford and BCA assays. The Bradford assay showed artificially elevated values for the lysis buffer blank, exceeding several standard curve points and patient samples. In contrast, the BCA assay produced expected results, with the blank reading below the lowest standard. This discrepancy likely stems from the known interference of SDS with Bradford reagents, while the BCA assay remains compatible with SDS-containing samples.

Standard (ng/µL)	(Abs 1)	(Abs 2)
**125**	0,358	0,353
**250**	0,351	0,346
**500**	0,408	0,458
**750**	0,624	0,603
**1000**	0,722	0,718
**1500**	0,978	0,941
**2000**	1,137	1,193
**Blank**	0,225	0,231
**Patient sample 1**	0,559	0,555
**Patient sample 2**	0,45	0,469
**Patient sample 3**	0,634	0,599
**H_2O**	0,204	0,215

Reviewer 1, Table 2. BCA.

Standard (ng/µL)	(Abs 1)	(Abs 2)
**125**	0,641	0,599
**250**	0,716	0,723
**500**	0,842	0,885
**750**	1,021	1,014
**1000**	1,149	1,102
**1500**	1,297	1,234
**2000**	1,499	1,45
**Blank**	1,019	1,025
**Patient sample 1**	0,921	0,941
**Patient sample 2**	0,958	0,953
**Patient sample 3**	0,975	0,977
**H_2O**	0,55	0,569

Reviewer 1, Table 3. Bradford.

5. *"The results are thorough, with clear visualizations supporting the text. However, Figure legends could provide more contextual explanations."*

We thank the reviewer for the positive comments on our figures. We appreciate the feedback on the legends and have now thoroughly revised all figure legends to better convey key messages while maintaining appropriate technical detail.

6. *"Some variability in TP and housekeeping proteins across patient samples is discussed, but it would be helpful to hypothesize more concretely on the biological factors contributing to these differences."*

We thank the reviewer for this important suggestion and have added a new paragraph to the discussion:

To our knowledge, this is the first study comparing TP and housekeeping protein normalization strategies in isolated primary human adipocytes. The substantial inter-individual variation in housekeeping protein expression observed in our study aligns with findings from other primary human tissue analyses: Similar variability has been reported in the human cortex (6), where actin displayed robust heterogeneity between individuals, while Ponceau TP staining provided more consistent normalization. In skeletal muscle, both GAPDH and actin demonstrated age-dependent expression decreases, while stain-free TP measurement remained stable for both inter- and intraindividual variation (8). Similarly, placental reference proteins exhibited significant variation while total protein remained stable (9). The biological basis for these differences likely stems from the dynamic nature of cellular metabolism. For example, metabolic enzymes such as GAPDH may vary with energy status (26,27), while structural proteins like actin could fluctuate with cell or tissue remodeling processes (28,29). These variations are particularly relevant in adipocytes, where protein expression can be influenced by factors such as adipose depot location, BMI, age or systemic factors. The consistency of these findings across multiple primary human tissues underscores the limitations of housekeeping protein normalization and supports the broader applicability of TP normalization in human tissue analysis.

7. *"Consider summarizing the key advantages of TP normalization over individual housekeeping proteins in a concise table or schematic for clarity."*

We thank the reviewer for this excellent suggestion and have added a new table, Table 1 (shown below), which provides clear guidance on optimal normalization references for different experimental designs.

Experimental setup	Best overall	Best housekeeping protein
**Technical replicates SC**	TP	FAH
**Technical replicates OM**	TP	Actin
**Dynamic range**	TP	Actin
**Inter-individual comparison**	TP	Actin

Table 1. Optimal Normalization References for Different Experimental Designs.

8. *"The discussion appropriately highlights the benefits of TP normalization. It would strengthen the manuscript to compare these findings more explicitly with studies employing other normalization methods in similar contexts (e.g., specific adipocyte studies)."*

We appreciate this suggestion and have expanded our discussion to include a broader comparative analysis in the same paragraph as highlighted in point 6.

9. *"Addressing the potential limitations of TP normalization due to tissue-specific tryptophan content is valuable. Consider discussing whether this limitation could extend to clinical applications or other experimental designs."*

We thank the reviewer for this insightful comment. We have now added a comprehensive discussion of tryptophan-related considerations in the discussion:

One limitation of stain-free technology is that it works by modifying tryptophan residues, which could affect TP quantification depending on the sample's tryptophan content. While tryptophan metabolism varies between individuals and species, studies suggest that tryptophan levels in the proteome remain consistent within individuals of the same species (18,19). Certain conditions, such as inflammation (20), cancer (21), kidney dysfunction (22), pregnancy (23), and treatments like interferon therapy (24) or indoleamine 2,3-dioxygenase 1 (IDO1) inhibitors (25), are associated with altered tryptophan metabolism. However, there is currently no evidence to suggest that these changes significantly impact the tryptophan content of cellular proteins or the abundance of tryptophan-containing proteins. In cases where changes in tryptophan-containing proteins are known or suspected, alternative TP normalization methods, such as Amido Black or Ponceau Red staining (which target proteins via general electrostatic and hydrophobic interactions), or the use of stable housekeeping proteins (Table 1), may provide more reliable results.

10. *"The supplementary figures are well-constructed, but it may be helpful to integrate key supplementary findings directly into the main figures for a more cohesive narrative. Ensure all figure captions are self-contained, explaining abbreviations and experimental conditions in detail."*

We appreciate this feedback and have made text adjustments in the figure legends to better accentuate key findings (in response to comment 5). After considerable clarifications to the main text, we believe the flow of information and messaging is significantly improved, without the need to move any supplemental data to the main manuscript. We would be happy to reconsider this if the reviewer or editor have any specific figure in mind?

11. *"The conclusion strongly advocates for TP normalization but could also acknowledge scenarios where housekeeping proteins might still be appropriate, particularly for specific experimental designs."*

The reviewer raises a good point, which we have now addressed through the addition of a new table, Table 1 (shown below), which includes a dedicated column for situations when TP is not

feasible ('Best housekeeping protein'). Additionally, our expanded discussion of BSA requirements and tryptophan metabolism provides clear guidance on when alternative normalization strategy may be more appropriate.

Experimental setup	Best overall	Best housekeeping protein
**Technical replicates SC**	TP	FAH
**Technical replicates OM**	TP	Actin
**Dynamic range**	TP	Actin
**Inter-individual comparison**	TP	Actin

Table 1. Optimal Normalization References for Different Experimental Designs.

12. *"Ensure consistency in terminology and abbreviations throughout the manuscript. Revisit the statistical analysis details to ensure they are sufficiently described, particularly regarding how variability was quantified across replicates and normalization references."*

We thank the reviewer for bringing this to our attention. We have thoroughly reviewed the manuscript for consistency in terminology and abbreviations. For readers interested in the methodology, Supplementary Table 1 provides a step-by-step calculation for technical replicates and dynamic range, with linked cells showing data origin, visualization graphs, and CV calculations. Additionally, we have enhanced the methods section with more detailed statistical information:

The coefficient of variation (CV) was calculated using the deviation values obtained from the equations above. The standard deviation was divided by the mean of the deviation values and multiplied by 100 to generate a percentage using Prism 9 software (GraphPad), quantifying the relative variability in protein expression across experimental conditions (6–10).

13. *"The ethics statement is clear, but more details on the demographic and clinical background of the patient cohort might enhance reproducibility and relevance."*

We thank the reviewer for this suggestion. We have included clinical information for all patient samples. Summaries can now be found in Supplementary Tables S3-4. While demographic information beyond the clinical parameters provided in Supplementary Table 3-4 is not available

due to privacy regulations and ethical constraints, we have included relevant metabolic measurement that we believe could impact the interpretation of our results. We believe these clinical parameters provide sufficient context for the reproducibility and interpretation of our findings regarding protein normalization methods. Additional text has been added to the methods section:

A summary of clinical and metabolic parameters of the full cohort can be found in S3-4 Tables.

Clinical parameters	Individual 1	Individual 2	Individual 3
**Depot**	OM	OM	OM
**Age**	47	50	46
**Sex**	Female	Female	Female
**BMI (kg/m²)**	37,41	37,73	38,12
**Fat (%)**	48,3	45,4	43,2
**Hip (cm)**	122,5	120	123
**Waist (cm)**	111	102	95
**WHR**	0,91	0,85	0,77
**Medication**	None	None	None
**Glucose**	5,7	5,8	5,7
**C-peptide**	0,99	1	1
**Insulin**	10	12	13

S3 Table. Clinical parameters for patient cohort used in Fig 4.

Clinical Parameters	BSA	OBHI OM	OBHI SC	OBHI OM	OBHI SC	Dynamic range
**Depot**	OM	OM	SC	OM	SC	OM
**Age**	52	53	63	29	53	37
**Sex**	Male	Female	Female	Female	Female	Female
**BMI (kg/m^2)**	38.51	38.38	42.05	34.05	33.94	43.47
**Fat (%)**	36.4	49	51.9	48.9	47.6	53.3
**Hip (cm)**	139	124	133	124	119	131
**Waist (cm)**	142	114	130	103	101	109
**WHR**	1.02	0.92	0.98	0.83	0.85	0.83
**Medication**	Metformin, Xultrophy	losartan, imraldi, dymista	lamictal, eliqwas, atorvastatin	bricanyl	Levaxin	Brintellix
**Glucose**	5.6	6.3	5.5	5.6	5.7	5.4
**C-peptide**	0.56	1.4	1.8	0.81	0.59	1.6
**Insulin**	12	24	42	14	8.5	30

S4 Table. Clinical parameters for all patient samples.

Reviewer 2

1. *"The authors in their studies clearly revealed that TP (Total Protein) normalization methods in western blotting are far superior to housekeeping (GLUT4), actin, and tubulin protein-based normalization methods, including BSA contaminations. The methods that they suggested are more reliable and cost-effective, as they are not using any antibodies in their work and are strain-free too."*

We thank the reviewer for their positive evaluation of our work and the recognition of the advantages of TP normalization in Western blotting

2. *"But studies were limited to only primary mature human adipocytes. No more comparisons/discussions with other cell types of human samples."*

We appreciate this insightful comment and agree that including a discussion on housekeeping protein expression in other human tissues would broaden the scope and appeal of our manuscript. We have added a paragraph addressing this point in the discussion:

To our knowledge, this is the first study comparing TP and housekeeping protein normalization strategies in isolated primary human adipocytes. The substantial inter-individual variation in housekeeping protein expression observed in our study aligns with findings from other primary human tissue analyses: Similar variability has been reported in the human cortex (6), where actin displayed robust heterogeneity between individuals, while Ponceau TP staining provided more consistent normalization. In skeletal muscle, both GAPDH and actin demonstrated age-dependent expression decreases, while stain-free TP measurement remained stable for both inter- and intraindividual variation (8). Similarly, placental reference proteins exhibited significant variation while total protein remained stable (9). The biological basis for these differences likely stems from the dynamic nature of cellular metabolism. For example, metabolic enzymes such as GAPDH may vary with energy status (26,27), while structural proteins like actin could fluctuate with cell or tissue remodeling processes (28,29). These variations are particularly relevant in adipocytes, where protein expression can be influenced by factors such as adipose depot location, BMI, age or systemic factors. The consistency of these findings across multiple primary human tissues underscores the limitations of housekeeping protein normalization and supports the broader applicability of TP normalization in human tissue analysis.

3. *"Noise eliminations and contaminations, like with BSA proteins, were discussed exhaustively, but no data was not shown for noise elimination or contamination removal."*

We thank the reviewer for raising this important point and prompting us to better highlight this information. The impact of contamination, such as BSA, is discussed in more detail in the revised

manuscript and data demonstrating the impact of removing contamination (BSA-free samples) is shown in Figure 1. In this figure, we compare the performance of total protein normalization versus the housekeeping protein tubulin in adipocytes isolated with and without BSA. Additionally, we investigated whether BSA contamination could be removed by washing BSA-isolated cells three times with PBS, which would enable accurate total protein normalization. Our findings demonstrate that while PBS washing substantially reduces BSA contamination, it does not eliminate it entirely. Consequently, we recommend avoiding exogenous proteins contaminations (such as BSA) altogether where possible and the use of housekeeping proteins for normalization when lysates contain any exogenous protein.

Reviewer 3

We thank the reviewer for their detailed critique of our manuscript. While we acknowledge their expertise and suggestions for broader protein profiling approaches, we believe that our focus on isolated adipocytes offers unique insights into best practices for western blotting in adipocytes. By responding to the questions bought up by this (and the other) reviewers, we believe the manuscript has been significantly strengthened and better highlights the contribution of this work to the field of adipocyte biology.

1. *"The concept of the manuscript is not particularly novel, as stain-free total protein normalization has been a common way of normalizing Western blot data for more than ten years. However, it could still be of particular interest if done rigorously and in a way where a generalizable improvement can be shown in adipocytes. In this study, there are, unfortunately, too many issues with the experimental design."*

While total protein normalization is indeed an established technique, our study makes a novel contribution by specifically addressing and validating its application in mature human adipocytes, where sample availability is limited, and donor variation high. The unique challenges of working with primary human adipocytes, including their fragility, high lipid content, and metabolic heterogeneity, necessitates a careful validation of normalization methods. We have expanded our discussion to better highlight the specific challenges and considerations when working with primary human adipocytes, including pros, cons and caveats of using total protein normalization. We have also provided a new comprehensive table (Table 1, shown below) that outlines the optimal normalization references for different experimental designs, including scenarios where TP normalization may, or may not, be most appropriate. This practical guidance will help researchers select the most suitable normalization strategy for their specific experimental conditions.

Table 1. Optimal Normalization References for Different Experimental Designs.

Experimental setup	Best overall	Best housekeeping protein
**Technical replicates SC**	TP	FAH
**Technical replicates OM**	TP	Actin
**Dynamic range**	TP	Actin
**Inter-individual comparison**	TP	Actin

2. *"i) The study relies on commercially available antibodies, and it is of utmost significance to ensure that the antibodies used in the experiments are thoroughly validated for the type of study performed. There is no discussion regarding the validation of the antibodies used,*

and there is only accessible validation data for the antibody targeting FAH (#PA5-42049). Additionally, this antibody is referred to as an anti-FAA antibody even though the target referenced by the vendors is FAH. There is no common gene abbreviation called FAA. This antibody is also disregarded due to signal saturation, which could easily be avoided by protocol optimization. A few recommended readings on antibody validation are:
- "Antibody validation" by Bordeaux et al. from 2010, BioTechniques.
- "Enhanced validation of antibodies for research applications" by Edfors et al. from 2018, Nature Communications.
- "Antibodypedia, a Portal for Sharing Antibody and Antigen Validation Data" by Björling and Uhlén from 2008, Molecular & Cellular Proteomics. "

We appreciate the reviewer's emphasis on the importance of antibody validation and acknowledge the error in referring to fumarylacetoacetate hydrolase (FAH) as fumarylacetoacetase (FAA). This has been corrected throughout the manuscript to align with the vendor's nomenclature. While "FAA" is occasionally used as a shorthand for FAH, we agree that consistent use of the term FAH is critical to avoid confusion.
Regarding antibody validation, we used commercially available antibodies that were validated by the manufacturers and recommended for use in western blotting. In our experimental system, these antibodies detected proteins at their expected molecular weights, supporting their specificity.
We agree with the reviewer that protocol optimization for each housekeeping antibody would be ideal, particularly to address signal saturation issues such as that observed with FAH. Our intent was not to disregard FAH as a housekeeping protein, but rather to highlight the need for optimization when working with housekeeping proteins. The primary aim of our study was to evaluate and compare housekeeping proteins as normalization references under standard western blot conditions. This approach allowed us to assess the technical performance of these normalization strategies, including identifying challenges such as FAH signal saturation.
While protocol optimization could potentially mitigate saturation for FAH, we faced practical constraints inherent to primary tissue research. Optimization for each antibody would require separate sample preparations for every adipose depot and experimental condition, which is not always feasible given the limited availability of primary human adipose tissue. New text has been added to the methods section, clarifying that the antibodies used in this study have been commercially validated:

Primary antibodies, <u>all commercially validated</u>, were prepared in 10 mL 5% milk (Millipore, #70166-500G) at the following concentrations; BSA (Life, 1:1000, #A11133), GLUT4 (ProteinTech, 1:500, #66846-1-Ig), PARK7 (Abcam, 1:500, #ab18257), ENOA (MyBioSource, 1:250, #MBS9231722) and FAH (Invitrogen, 1:500, #PA5-42049). After primary antibody incubation, the membrane was washed 3 times in TBS supplemented with 0,05% tween-20 (TBST) (ChemCruz, #SC-362311) and incubated with secondary antibody for 1 hour at RT.

3. *“ii) The authors only investigate the normalization in the context of one protein target. The lack of different protein targets subjected to normalization nullifies any generalization of the findings, and making any generalized claims without investigating at least a smaller data set of ten different target proteins would be ill-advised.”*

While we appreciate the value of a broader protein target panel for large-scale protein profiling, the main focus of our study was to compare housekeeping proteins with total protein, to investigate any systematic limitations of housekeeping protein normalization in primary mature human adipocytes. Our conclusions regarding the superiority of total protein normalization remain valid, independent of the number of target proteins, as demonstrated by our analysis:

1. Our investigation revealed that total protein typically demonstrates superior stability compared to housekeeping proteins in technical replicates, independent of any target protein normalization. As demonstrated by our analysis, the CV for housekeeping proteins was higher compared to TP in most cases (Fig 2A-F, S3A-F Fig, left-side panels), with only two exceptions: FAH showed the lowest CV for the OBHI OM sample, and actin had the lowest CV for the OBNI OM sample (Fig 2D, S3F Fig). Importantly, even when housekeeping proteins showed lower variance, different proteins performed best under different metabolic conditions, highlighting the challenge of selecting a consistently reliable housekeeping protein.

2. The dynamic range analysis revealed an inherent limitation in housekeeping protein reliability, such as signal saturation observed with FAH at protein loads above 7.5 µg (Fig 3C and S5C Fig). In contrast, TP maintained linearity across the entire tested range and was the only normalization reference with a CV consistently below 10% (Fig 3A-C).

3. We demonstrated substantial inter-individual variation in housekeeping protein expression (2-6-fold differences) even amongst metabolically similar patients (Fig 4D-F). This inherent variability in housekeeping protein expression would affect normalization of any target protein, not just GLUT4.

Together, these findings demonstrate fundamental limitations in housekeeping protein stability that would affect normalization of any target protein. These inherent variabilities in the normalization references themselves support our conclusions about TP normalization's superiority, independent of the number of proteins analyzed.

4. *“iii) The manuscript does not have a proper statistical evaluation that supports the claim of improved accuracy with total protein normalization, and there are no established*

reference values for the samples that can indicate if the observed intensities actually reflect the truth."

Our use of coefficient of variation (CV) analysis provides a robust and widely accepted method for assessing measurement stability. This approach is well-documented in the literature, with multiple studies using CV to assess housekeeping and total protein options for western blot analysis (PMID: 30367480, 26004848, 25429098, 22244735, 22272336). This statistical approach is particularly appropriate for our experimental context, as we aim to quantify and compare the stability of different normalization methods across technical replicates, dynamic range and patient samples.

Furthermore, while some studies have used Pearson correlation to evaluate normalization method performance in dynamic range experiments, we contend that correlation analysis alone is insufficient. A high correlation coefficient can mask systematic deviations from expected values, as a protein signal might show strong linearity while failing to scale proportionally with sample loading. Our normalization approach addresses this limitation by calculating the fraction of total intensity for each band/lane and comparing it to the expected fraction (see Methods). This method not only assesses linearity but also ensures proper scaling of signal intensity with protein load (see figure below). This more comprehensive analysis provides a better assessment of normalization reference reliability across the full dynamic range of protein loading (plots and table included for reviewer only).

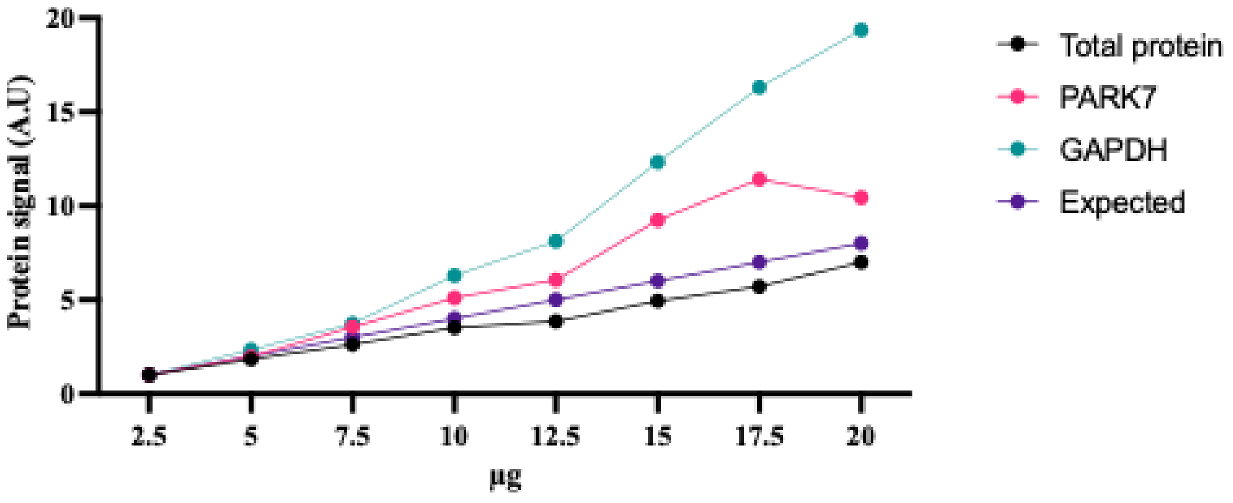

Reviewer 3, Figure 1. Pearson correlation analysis of the dynamic range for TP, PARK7 and GAPDH, compared to expected values. While both PARK7 and GAPDH show high R^2 values, they deviate significantly from the expected values, indicating a poor fit despite the strong correlation. To accurately assess performance, CV is a better metric (reviewer table 1). For total protein CV and R^2 values both show good fit.

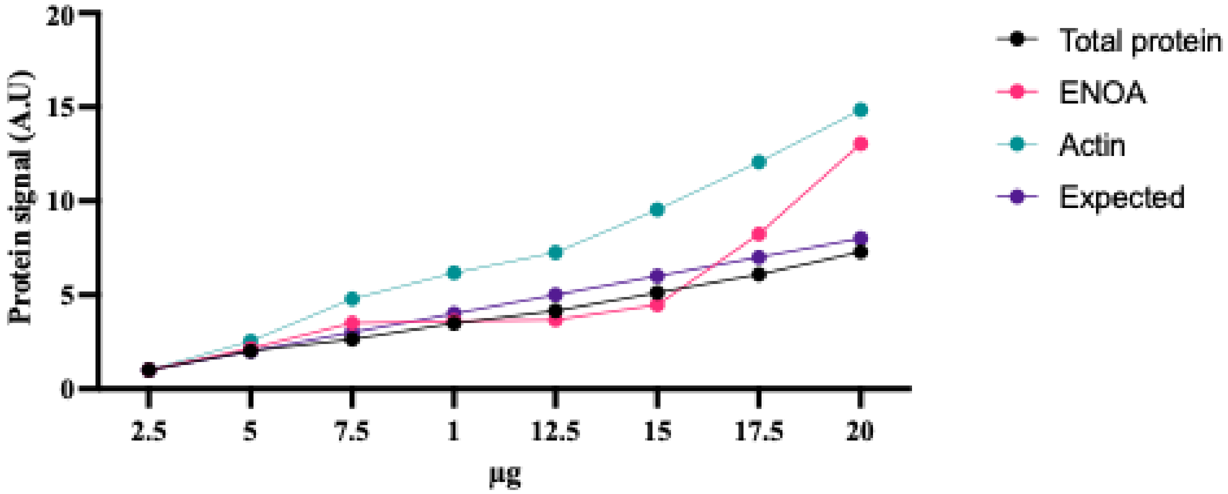

Reviewer 3, Figure 2. Pearson correlation analysis of the dynamic range for TP, ENOA, and Actin compared to expected values. For ENOA, both the R^2 value and CV indicate that its dynamic range is poor and does not align with the expected values. Actin shows a high R^2 but a high CV, reflecting its deviation from the expected values. In contrast, total protein demonstrates both low CV and a high R^2, indicating a robust fit to the expected values (reviewer table 1).

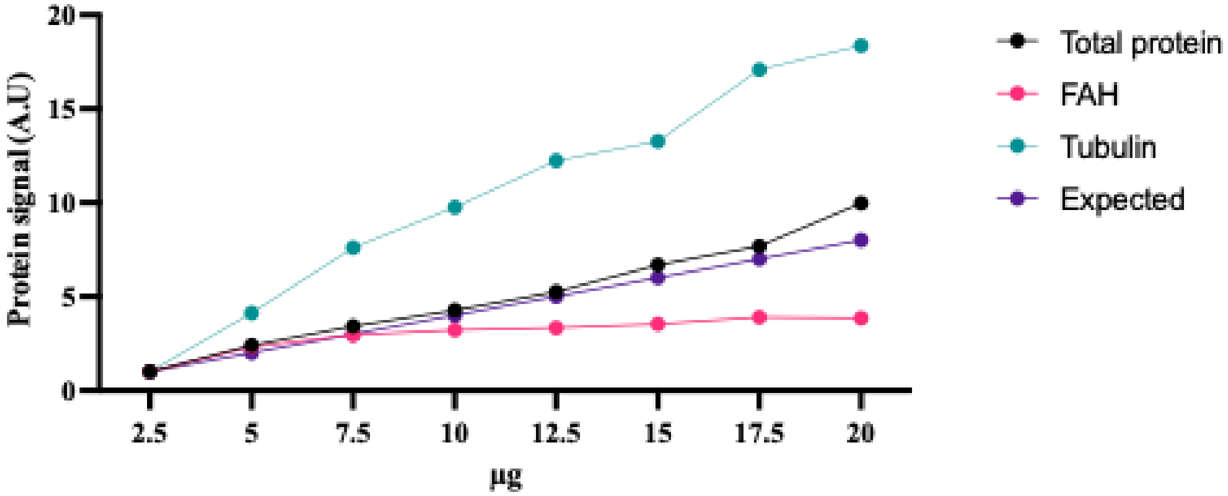

Reviewer 3, Figure 3. Pearson correlation analysis of the dynamic range for TP, FAH, and Tubulin compared to expected values. For FAH, both R^2 and CV confirm that its dynamic range is suboptimal and does not align well with the expected values. Tubulin, while having a high R^2, exhibits a high CV, indicating substantial deviation from the expected values. In contrast, total protein shows strong agreement with expected values, as reflected by both low CV and high R^2.

Normalization reference	R^2	CV
**Total protein (Blot 1)**	0.9897	8.3
**PARK7**	0.9543	16.9
**GAPDH**	0.9642	27.9
**Total protein (Blot 2)**	0.9929	7.6
**ENOA**	0.7814	26.2
**Actin**	0.9842	16.5
**Total protein (Blot 3)**	0.9833	7.1
**FAH**	0.8144	26.7
**Tubulin**	0.9862	21.6

Reviewer 3, Table 1. R^2 values and CV's for all normalization references for all dynamic range blots.

While GAPDH, actin, and tubulin display high linearity, their measured values strongly deviate from expected levels, which is not adequately captured by the R^2 value. To address this limitation, we opted to use CV for a more accurate assessment.

To enhance clarity, we have expanded the methods section with more detailed information about our statistical analyses, specifically describing how CV calculations were performed and used to assess variability:

The coefficient of variation (CV) was calculated using the deviation values obtained from the equations above. The standard deviation was divided by the mean of these deviation values and multiplied by 100 to generate a percentage using Prism 9 software (GraphPad), quantifying the relative variability in protein expression across experimental conditions (6–10).
