# Supplementary material for: Superior normalization using total protein for western blot analysis of human adipocytes
Source: PLoS One. 2025 Jul 22;20(7):e0328136. doi: 10.1371/journal.pone.0328136 (PMC12282925; doi:10.1371/journal.pone.0328136)
Supplement: Attachment — Submitted filename: Response to reviewers Westerberg et al..pdf [file pone.0328136.s015.pdf]

Rebuttal PONE-D-24-50811R1
"Superior normalization using total protein for western blot analysis of human adipocytes"

June 18, 2025
Stockholm, Sweden

Dear Editor and Reviewers,

Thank you for taking the time to review our work and for your thoughtful feedback.

Please find below our point-by-point response to comments (author response in blue) as well as our response to the general comments requested of the reviewers.

All line references refer to the clean version of the manuscript.

Thank you again for this constructive review process, we believe the manuscript has been substantially strengthened as a result.

Kind regards,
Kirsty

Kirsty Spalding
Professor | Vice-Chair Department of Cell and Molecular Biology
Wallenberg Scholar

Karolinska Institutet
Biomedicum B7 | Solnavägen 9 | SE-171 65 Solna
Aula Medica | Nobels väg 6 | Floor 7 | SE-171 77 Stockholm
Phone: +46 (0)70 437 1542
E-mail: kirsty.spalding@ki.se

Karolinska Institutet – a medical university

Reviewer 1
I have gone through with the revised manuscript and found it suitable for the publication. Authors have addressed all comments.

We thank you for your positive evaluation and constructive comments, which we believe have helped improve the quality and clarity of our work.

Reviewer 3
1. The authors seem to have misunderstood the comment regarding assessing normalization in multiple target proteins. In the study, normalization is only evaluated in the context of GLUT4. To claim that the normalization is superior for experiments conducted in primary human adipocytes, the normalization strategies must be assessed for multiple proteins of variable expression between individuals. Without this comparison, the claims of superior normalization can only be applied to GLUT4 specifically.

Response to Reviewer 3, Comment 1
Thank you for this important point. We agree that demonstrating the generalisability of our normalization strategy beyond a single protein target strengthens our conclusions. To address this, we have:

(i) **Clarified the role of GLUT4** as an illustrative example, not the sole basis for our claims.
(ii) **Added a simulation-based analysis** to show how reference-protein variability affects normalized measurements irrespective of the target protein.

We would like to clarify that GLUT4 was included merely as a "real-world" example of our workflow, not to imply that our conclusions apply only to GLUT4. In practice, the benefits of total-protein normalization, namely, its superior technical reproducibility, increased inter-individual stability, and broader dynamic range, are independent of the particular protein being measured.
To illustrate this universality, we generated simulated Western-blot datasets with controlled variability. Using the Box-Muller transformation, we created a "protein of interest" (POI) dataset with a realistic technical CV of 19.9% (mean = 1) and three randomized independent "reference" datasets with CVs of 5%, 15%, and 30%. We then normalized the POI values to each reference and recalculated CVs.

(i) **Stable reference (5% CV):** Normalization maintained high precision (final CV = 16.6%), closely matching the POI's original variability.
(ii) **Moderately variable reference (15% CV):** Normalization inflated variability (final CV = 30.6%).
(iii) **Highly variable reference (30% CV):** Normalization further increased variability (final CV = 47%), a 2.8-fold loss of precision compared to the stable reference (16.6% stable vs 47% highly variable).

These results (now shown in the revised manuscript's Methods, starting line 199; Results, starting line 210; and New S2 Fig.) demonstrate that reference-protein stability directly governs the precision of normalized measurements, regardless of which target protein is analyzed. Importantly, all simulations assume identical loading and isolate only the

effects of reference variability on quantification precision. While biological factors (e.g., correlations between target and reference expression) are not accounted for in the model here, our findings establish a general analytical principle: total-protein normalization minimizes technical noise and maximizes reproducibility across any protein target.

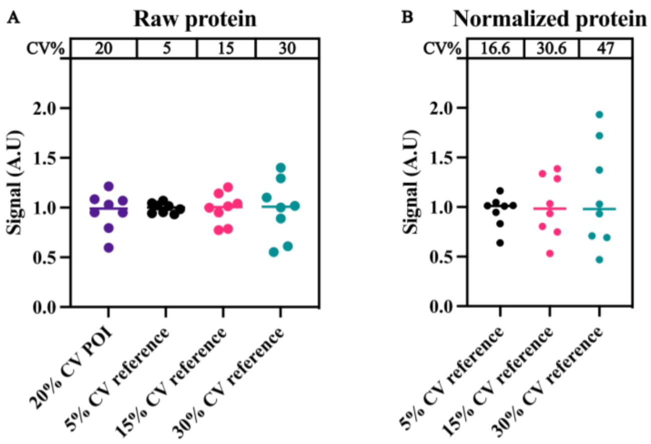

New S2 Fig. Simulation of Western-blot normalization via seeded random data. (A) Raw signal intensities for a protein of interest (POI) and three normalization references with increasing coefficients of variation (CV = 5 %, 15 %, 30 %). (B) Normalized POI signals (POI/reference) for each CV level. As reference variability rises, normalized-signal stability deteriorates, even though the POI's underlying variability remains constant.

2. Additionally, there is a significant need to discuss the validation of the antibodies used beyond "all commercially validated" when using these as the basis for comparing different normalization strategies. As stated in the previous reviewer comments, there is only publicly available validation data for one of the antibodies, and a discussion and justification for the choice of these antibodies should be included in the manuscript.

Response to Reviewer 3, Comment 2
We appreciate the reviewer's emphasis on rigorous antibody validation. Below, we summarise both published evidence and our in-house checks for each antibody used (we are happy to include images from all referenced figures in our rebuttal if the editor/reviewer wishes):

GLUT4 antibody (66846-1-Ig, Proteintech)

- **Literature support:** Cited in >90 peer-reviewed studies for Western blot (WB) and immunocytochemistry (ICC) with consistent detection of the 45–55 kDa GLUT4 band (see list: https://www.citeab.com/antibodies/6638026-66846-1-ig-glut4-monoclonal-antibody?des=3de7421913174b6d).
- **Manufacturer data:** Raised against AA 1–100 of human GLUT4; minimal cross-reactivity to other GLUT isoforms (datasheet).

PARK7 antibody (ab18257, Abcam)

- **Vendor validation:** Abcam's KO-cell analysis shows complete signal loss in PARK7 KO HAP1 cells (https://www.abcam.com/en-us/products/primary-antibodies/park7-dj1-antibody-ab18257).
- **Cross-species:** Validated on human, mouse, and rat lysates.

FAH antibody (PA5-42049, Thermo Fisher)

- **Knockdown validation:** Demonstrated in DOI:10.1002/mgg3.2090, Fig. 3A.

Housekeeping-protein antibodies

- **GAPDH (12004167), Actin (12004163), Tubulin (12004165):**

Literature usage: Each antibody has been used extensively (GAPDH: 25 studies; Actin: 50; Tubulin: 44, see https://www.citeab.com/...), consistently yielding single bands at expected weights.

Practical constraints: True KO/knockdown is often not feasible for essential proteins; instead, we rely on extensive community usage and concordant fractionation data (e.g., purity markers in cytosolic vs. nuclear fractions).

ENO1 antibody (MBS9231722, MyBioSource)

- **Overexpression validation:** Shown in Blood 2008 (10.1182/blood-2008-08-170837, Fig. 5A).

While we have selected antibodies with strong literature precedent, we recognise that no single antibody validation is perfect. This limitation further motivates our adoption of total-protein normalization: it minimises dependence on any one antibody's specificity and provides a more robust, reproducible baseline across experiments.

3. As the authors state in their response, they have done a robust statistical assessment of the measurement stability. Still, stability is not the same thing as accuracy. Without any established reference values for GLUT4 in the different samples from individuals (preferably with a different methodology), there is no evidence of improved accuracy in the measurements. Accuracy is a measurement of how well the quantification reflects the actual amount of the analyte. Accuracy can, for example, be assessed by measuring the samples with an orthogonal method or by spiking a sample with different amounts of a recombinant protein. This is of significant importance when working with normalization strategies, as the normalization directly impacts the measurement values obtained from an analysis.

Response to Reviewer 3, Comment 3

Thank you for this insightful point. We apologise for any lack of clarity regarding the scope of our analysis. Our goal was not to determine the absolute abundance of GLUT4 in adipocyte lysates, but rather to use GLUT4 as a representative "real-world" example to illustrate how the choice of normalization reference, particularly different housekeeping proteins, can dramatically influence the precision of normalized protein measurements across individuals.

To make this explicit, we have:

1. **Emphasised the illustrative nature of GLUT4**

We have revised the text to state clearly that GLUT4 serves only as an example target. Our aim is to highlight variability in normalization references, not to benchmark GLUT4 quantification accuracy per se.

2. **Linked back to our simulation analysis**

As detailed in our response to Comment 1, we now include simulated Western-blot datasets showing that, even with identical underlying protein signals, increasing variability in the reference inflates normalized-signal imprecision. This controlled demonstration (New S2 Fig) underscores that reference stability, not the target protein, drives normalized-measurement precision.

3. **Clarified Figure 4's purpose**

We have updated the legend (line 386) and main text to specify that Figure 4D-F illustrates inter-individual variability of six housekeeping proteins, before and after total-protein normalization. These panels are intended to show that:

- Normalizing housekeeping proteins to total protein only partly reduces their inter-patient variability.
- Different housekeeping proteins display distinct variability profiles across donors.
- Such variability violates the assumption that a normalization reference is stable and independent of biological conditions.

We agree that evaluating **accuracy** (i.e., closeness to the true protein amount) would require orthogonal methods or spike-in standards. While this is beyond the present study's scope, our simulation and real-data analyses together establish a compelling case that total-protein normalization minimizes technical noise and yields more reproducible quantification, an essential prerequisite for accuracy in any downstream measurement. We have made these clarifications in the Methods section (line 193), the Results section (line 423), and the Discussion section (line 530) of the revised manuscript to ensure that the distinction between precision (measurement variability) and accuracy (true-value agreement) is fully transparent.

Reviewer 4:

1. Lines 246-253 "Overexposure of the blot showed that the expected GLUT4 signal (~54 kDa) was clearly present in the BSA-free samples while the BSA-isolated samples showed non-specific bands at ~60 kDa (S2B Fig). These results can be explained by anti-GLUT4 antibody binding non-specifically to BSA (65 kDa) leading to false positives in BSA-isolated samples but not in BSA-free samples. Thus, subsequent experiments within this study used BSA-free samples, accentuating the importance of limiting BSA, or other protein contaminants, in western blotting". Is there a reason why the authors speculate that is the anti-GLUT4 that is cross-reacting with BSA and not the secondary antibody?

Response to Reviewer 4, Comment 1

Thank you for pointing this out. You are correct that we cannot definitively attribute the cross-reactivity to the primary versus the secondary antibody without additional experiments. To avoid overinterpretation, we have revised the text (Lines 279–285) as follows:

"Overexposure of the blot showed that the expected GLUT4 signal (~54 kDa) was clearly present in BSA-free samples, while BSA-isolated samples exhibited a non-specific band at ~60 kDa (S2B Fig). These results can be explained by non-specific antibody binding to BSA (65 kDa), leading to false positives in BSA-isolated samples but not in BSA-free samples. Thus, subsequent experiments within this study used BSA-free samples, accentuating the importance of limiting BSA, or other protein contaminants, in western blotting."

This wording removes any implication about which antibody is responsible, accurately reflecting our observations. Thank you for helping us clarify this point.

2. I am curious if is there a technical/biological reason for GLUT4 selection as target gene? Notoriously, WB of membrane proteins are less clean and often characterised by double bands not very sharped associated to the glycosylated and non-glycosylated forms.

Response to Reviewer 4, Comment 2

Thank you for this thoughtful question. GLUT4 was chosen purely as a methodological test case because, in our hands, it yields robust, clear, and highly reproducible bands under our optimised western-blot protocol for mature human adipocytes. We acknowledge that membrane proteins often display glycosylation-related doublets or diffuse bands; however, GLUT4 consistently produces interpretable signals across multiple donor samples, making it well suited for evaluating normalization strategies.

Importantly, the intent was not to draw any biological conclusions about GLUT4 regulation, but rather to use a realistic adipocyte protein target to benchmark how different normalization approaches perform.

3. Figure 2 shows technical replicates from OBNI individuals. Do I understand correctly that each dot represent different runs from the same lysate sample? If so, please add/reiterate this when you describe the results and in the figure legend.

Response to Reviewer 4, Comment 3

Thank you for the suggestion. We have clarified both the figure legend and the Results text to make this explicit:

Figure 2 legend (line 316) now reads:

"Each dot represents one well - a technical replicate of the same lysate sample run in separate lanes."

Results section (line 292) now changed to read: "First, the assessment involved technical replicates, where each well in a gel was loaded with 10 µg of protein from the same sample. Samples were obtained from four different individuals: two obese hyperinsulinemic (OBHI) patients (one subcutaneous (SC) and one omental (OM)) and two obese normoinsulinemic (OBNI) patients (one SC and one OM). Each sample was analyzed across three separate blots to assay all housekeeping proteins, allowing for the normalization of GLUT4 levels to two housekeeping proteins and TP within each blot."

We have made the analogous update to **S4 Fig** legend (line 655) for consistency.

4. I do not see a callout for table 1.

Response to Reviewer 4, Comment 4
Table 1 is cited twice in the current version of the manuscript: first on line 427 and again on line 498. It is initially referenced in the final paragraph of the Results section—not to present new data, but to provide a summary of the advantages and disadvantages of different normalization references across various experimental designs. We acknowledge that, because the table is introduced relatively late and does not contain original data, the callout may be easily overlooked. However, we believe that its current placement is appropriate and serves its intended purpose within the manuscript structure.

5. Sometimes µg was reported as ug (e.g., line 263, line 309). Please correct.

Response to Reviewer 4, Comment 5
We appreciate your attention to detail and have corrected all instances of "ug" to "µg" throughout the manuscript.

6. Sometimes numbers were reported with comma (e.g., 2,5 – line 300), sometimes with dot (e.g., 27.3, line 320). I suggest using everywhere the dot format.

Response to Reviewer 4, Comment 6
We appreciate the reviewer's attention to consistency and have replaced all instances of numbers reported with a comma to the dot format throughout the manuscript.

General questions from the journal:

1. If the authors have adequately addressed your comments raised in a previous round of review and you feel that this manuscript is now acceptable for publication, you may indicate that here to bypass the "Comments to the Author" section, enter your conflict of interest statement in the "Confidential to Editor" section, and submit your "Accept" recommendation.
Reviewer #1: All comments have been addressed

Reviewer #3: (No Response)
Reviewer #4: (No Response)

No response needed

2. Is the manuscript technically sound, and do the data support the conclusions?
The manuscript must describe a technically sound piece of scientific research with data that supports the conclusions. Experiments must have been conducted rigorously, with appropriate controls, replication, and sample sizes. The conclusions must be drawn appropriately based on the data presented.
Reviewer #1: **Yes**
Reviewer #3: **No**
Reviewer #4: **Yes**

We respectfully refer to our detailed point-by-point responses to Reviewer #3.

3. Has the statistical analysis been performed appropriately and rigorously?
Reviewer #1: **Yes**
Reviewer #3: **No**
Reviewer #4: **Yes**

In response to Reviewer #3: We note that one reviewer remains concerned about our analytical approach for comparing total protein and housekeeping-protein normalization in Western blot analysis. We therefore provide additional context demonstrating that our methodology for assessing normalization-reference suitability aligns with well-established standards across multiple independent studies:

Housekeeping-protein versus total-protein CV analysis
A commonly used approach for evaluating normalization reference performance uses coefficient of variation (CV) as the primary statistical metric to assess variability and stability:
• Hu et al. (2016) used CV analysis to evaluate seven housekeeping proteins, concluding that references with lower CVs represent superior normalization controls.
• Parkin et al. (2019) applied CV-based statistical assessment to β-actin and total protein stability across 194 human cortex samples, using CV as the determining factor for reference suitability.
• Vigelsø et al. (2015) assessed normalization references in human skeletal muscle using CV values as the quantitative stability measure.

Technical-replicate assessment
Our technical replicate analysis methodology similarly follows established statistical protocols for normalization assessment:
• Gürtler et al. (2013) performed technical-replicate analysis with CV calculations to evaluate Stain-Free total-protein detection stability.
• Collins et al. (2015) applied identical CV-based replicate variability analysis in cerebrospinal fluid samples.

• Lanoix et al. (2012) employed an intra-group, stability-based CV framework to assess reference protein stability in human placental tissues.

Dynamic-range testing:
Our dynamic range testing follows standard statistical approaches for normalization evaluation:
• Eaton et al. (2013) conducted protein gradient loading experiments with linearity assessment and CV calculations to determine reference performance across loading ranges.
• Aldridge et al. (2008) used systematic loading gradients paired with CV analysis to evaluate normalization reference performance over dynamic protein concentrations.

All of these studies validate CV as an appropriate metric for evaluating normalization controls in Western blot experiments. Our analysis exactly follows these established protocols, applied here for the first time in isolated primary mature human adipocytes, to ensure rigorous, reproducible quantification. We trust this clarifies that our statistical approach is both appropriate and common practice in the field.

4. Have the authors made all data underlying the findings in their manuscript fully available?

The PLOS Data policy requires authors to make all data underlying the findings described in their manuscript fully available without restriction, with rare exception (please refer to the Data Availability Statement in the manuscript PDF file). The data should be provided as part of the manuscript or its supporting information or deposited to a public repository. For example, in addition to summary statistics, the data points behind means, medians and variance measures should be available. If there are restrictions on publicly sharing data—e.g. participant privacy or use of data from a third party—those must be specified.
Reviewer #1: **Yes**
Reviewer #3: **Yes**
Reviewer #4: **Yes**

No response needed

5. Is the manuscript presented in an intelligible fashion and written in standard English?

PLOS ONE does not copyedit accepted manuscripts, so the language in submitted articles must be clear, correct, and unambiguous. Any typographical or grammatical errors should be corrected at revision, so please note any specific errors here.
Reviewer #1: **Yes**
Reviewer #3: **Yes**
Reviewer #4: **Yes**

No response needed
